# m^5^C‐Modified tRF3b‐Cys^GCA^‐23 Suppresses Bladder Cancer Malignancy by Repressing H3K18 Lactylation via Stabilizing RBM4

**DOI:** 10.1002/advs.202522294

**Published:** 2026-02-27

**Authors:** Xiaoling Ying, Yapeng Huang, Jian Huang, Qinyu Cai, Danni Zhang, Cong Chen, Yuxi Nie, Baotong Yang, Chuan Li, Wenyu Hu, Chang Xiong, Chengcheng Zhang, Ding Ji, Yaomin Liang, Mei Yang, Wenqi Wu, Weidong Ji

**Affiliations:** ^1^ Department of Urology The Second Affiliated Hospital of Guangzhou Medical University Guangzhou China; ^2^ Guangdong Provincial Key Laboratory of Urology Guangzhou China; ^3^ Center for Translational Medicine The First Affiliated Hospital Sun Yat‐sen University Guangzhou China; ^4^ The First Affiliated Hospital Jinan University Guangzhou China; ^5^ Department of Urology Affiliated Hospital of Guangdong Medical University Zhanjiang China; ^6^ Department of Cardiology Guangdong Provincial People's Hospital Guangzhou China; ^7^ Department of Otolaryngology The First Affiliated Hospital Sun Yat‐sen University Guangzhou China; ^8^ Department of Breast Surgery Zhujiang Hospital Southern Medical University Guangzhou China

**Keywords:** bladder cancer, Histone lactylation, m^5^C‐tRF3b‐Cys^GCA^‐23 (mtRC), NSUN6, RBM4

## Abstract

The epigenetic modification of transfer RNA (tRNA) and tRNA‐derived small RNAs (tsRNAs) is associated with the initiation and development of cancer. However, the biological role of m^5^C‐modified tsRNAs, especially in bladder cancer (BC), and their regulatory mechanisms remain unclear. Here, we identify a novel m^5^C‐modified tsRNA, m^5^C‐tRF3b‐Cys^GCA^‐23 (mtRC), whose expression is significantly downregulated in both tumor tissues and urine samples of BC patients and is strongly negatively correlated with the malignant progression of bladder cancer. In vitro and in vivo functional experiments reveal that mtRC, but not its unmodified counterpart (tRC), exhibits a tumor‐suppressive role. Furthermore, NOP2/Sun RNA methyltransferase 6 (NSUN6) regulates mtRC abundance and suppresses cell proliferation. Mechanistically, mtRC directly binds the oncosuppressor protein RNA‐binding motif 4 (RBM4) and improves its stability by preventing RBM4 ubiquitination, thereby upregulating RBM4 protein levels. RBM4 reduces the levels of glycolytic genes and decreases glycolysis, thereby inhibiting histone H3 lysine 18 lactylation (H3K18la). This reduction in H3K18 lactylation attenuates the transcriptional activation of the downstream oncogenes IL1RAP and VASH2, thereby ultimately suppressing tumor malignancy in BC. Together, our results not only underscore the critical role of mtRC in BC but also unravel a novel and coherent regulatory signaling axis—mtRC/RBM4/H3K18la/IL1RAP&VASH2—that orchestrates BC malignancy, suggesting mtRC may serve as a candidate therapeutic target for BC treatment.

## Graphical abstract

In this study, we identified an NSUN6‐dependent m5C‐modified tsRNA, m^5^C‐tRF3b Cys^GCA^‐23 (mtRC), that is downregulated in BC and inversely correlated with disease progression. Mechanistically, mtRC suppresses BC malignancy by stabilizing RBM4, attenuating glycolysis, and thereby limiting H3K18 lactylation–mediated activation of IL1RAP and VASH2.

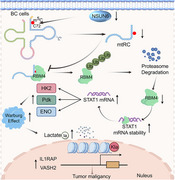

## 1 Introduction

tRNA‐derived small RNAs (tsRNAs) are generated by site‐specific cleavage of mature or precursor tRNAs and have emerged as important regulators [1]. These tsRNAs are categorized into subtypes based on the tRNA cleavage position: tRNA‐derived fragment (tRF)‐1, tRF‐2, tRF‐3, tRF‐5, and tRNA halves (tiRNAs) [2]. Additional evidence indicates that tsRNAs are involved in diverse biological roles, such as gene regulation, translation, and epigenetic regulation, and have been associated with various human diseases, including cancers [3, 4, 5, 6, 7].

To date, over 170 distinct post‐transcriptional RNA modifications have been documented, among which tRNA has the highest abundance of epigenetic modifications, such as methylation, pseudouridylation, and acetylation [8, 9, 10, 11, 12]. Because tsRNAs are generated from tRNAs, they inherit many of these modifications. Notably, many functional studies have relied on synthetic, unmodified tsRNA mimics, potentially underestimating how endogenous modifications influence base pairing, structural properties, and RNA‐protein interactions.

Consistent with this view, chemical modifications can endow tsRNAs with biological activities distinct from those of their unmodified counterparts [13]. For example, endogenous cytosine‐5 methylation (m^5^C)‐modified tsRNAs have been linked to metabolic disorders in offspring [14]. In addition, m^1^A‐ modified tRF‐3b exhibits reduced gene silencing activity [15], whereas pseudouridine (Ψ)‐ modified tRFs preferentially inhibit protein translation compared with their unmodified counterparts [16]. Similarly, 5‐methyluridine (m^5^U)‐ modified 3′‐t‐half mimics exhibit increased cytotoxicity [17]. Our previous research demonstrated that m^7^G ‐modified 3′ tiRNA‐Lys^TTT^, but not its unmodified counterpart, promotes bladder carcinogenesis [18]. Together, these findings highlight tsRNAs epitranscriptomics as a functional layer of regulation, although the underlying mechanisms remain incompletely understood.

Among the various tRNA modifications, m^5^C is widespread and occurs at multiple cytosine residues in mammalian tRNAs, including that at position 72 (C72) [19, 20, 21]. NSUN6 installs m^5^C specifically at position 72 in tRNA‐Cys (GCA) and tRNA‐Thr (CGU/GGU/UGU) [22]. While NSUN6‐dependent m^5^C modification of tRNAs has been characterized, the biological roles of m^5^C‐modified tsRNAs, particularly in bladder cancer (BC) pathogenesis, are poorly understood.

Histone lactylation is a recently identified metabolic and epigenetic modification driven by intracellular lactate and associated with transcriptional activation [23]. Given that aerobic glycolysis promotes lactate accumulation in tumors, histone lactylation has been implicated in cancer progression [24, 25]. However, upstream epitranscriptomic signals regulating site‐specific histone lactylation remain largely unknown.

Herein, we identified an NSUN6‐dependent m^5^C‐modified tsRNA, m^5^C‐tRF3b‐Cys^GCA^‐23 (mtRC), whose abundance is reduced in BC and negatively correlated with BC progression. We demonstrate, for the first time, that mtRC inhibits BC malignancy by stabilizing RBM4, dampening glycolysis, and consequently limiting H3K18‐lactylation‐dependent activation of IL1RAP and VASH2. These results provide a mechanistic link between tsRNA epitranscriptomic regulation and metabolic‐epigenetic transcriptional control in BC.

## 2 Results

### 2.1 Identification of a Novel Down‐Regulated m^5^C‐Modified tsRNA, m^5^C‐tRF3b‐Cys^GCA^‐23, in Transformed Urothelial Cells and BC Cells

**FIGURE 1:**
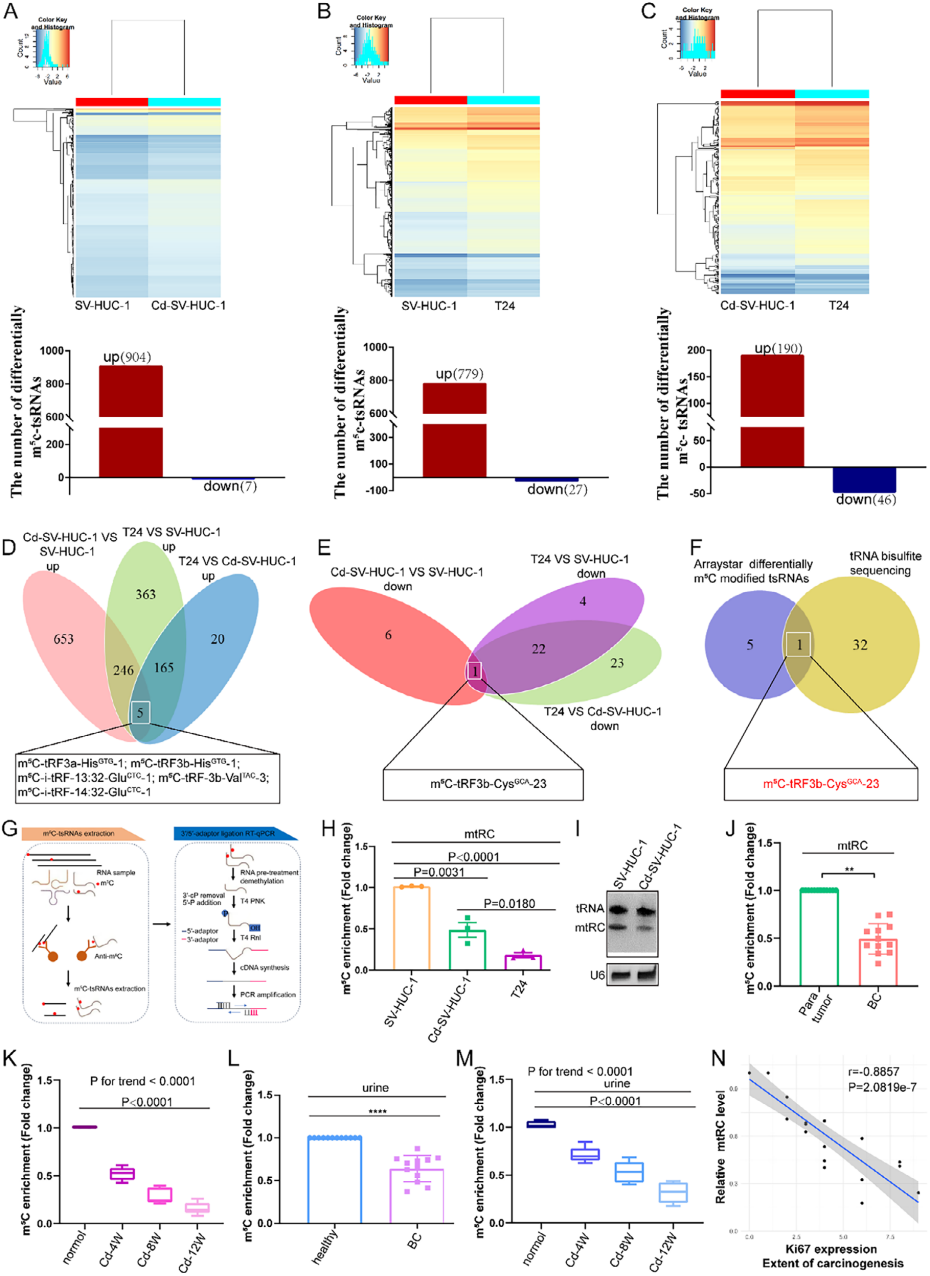
Identification of the novel m5C‐modified tsRNA m^5^C‐tRF3b‐CysGCA‐23 (mtRC) in transformed urothelial cells and bladder cancer (BC) cells. (A‐C) Heatmaps showing differentially expressed m^5^C‐tsRNAs identified by Arraystar Human m^5^C small RNA modification microarray in SV‐HUC‐1 vs. Cd‐SV‐HUC‐1 (A), SV‐HUC‐1 versus T24 (B), and Cd‐SV‐HUC‐1 vs. T24 (C). (D, E) Venn diagrams showing five commonly upregulated (D) and one commonly downregulated (E) m^5^C ‐tsRNAs across the three comparisons. (F) Venn diagram showing the intersection between tRNAs containing m^5^C modification (from tRNA bisulfite sequencing) and the six commonly regulated m^5^C ‐tsRNAs. (G) Flow chart of mtRC detection using m^5^C‐MeRIP‐3′/5′‐adaptor ligation RT‐PCR Method. (H) mtRC levels in SV‐HUC‐1, Cd‐SV‐HUC‐1, and T24 cells. Statistical significance was determined by one‐way ANOVA followed by Tukey's multiple comparisons test. (I) Representative Northern blot assay confirming mtRC expression in SV‐HUC‐1 and Cd‐SV‐HUC‐1 cells. (J) The abundance of mtRC was detected in BC tissues (*n* = 12) compared to para normal tissues (*n* = 12). Statistical significance was determined by two‐tailed paired t‐test. (K) The abundance of mtRC was detected in CdCl_2_ ‐induced multi‐stage bladder tissues and normal tissues (*n* = 5 rats per group). (L) mtRC expression was quantified in urine specimens from BC patients (*n* = 12) and healthy donors (*n* = 12). Statistical significance was determined by two‐tailed unpaired t‐test. (M) The abundance of mtRC was detected in multi‐stage urine specimens from previously established multi‐stage rat bladder cancer (*n* = 5 rats per group). (K,M) Data are presented as box plots showing the median and quartiles. Statistical significance was determined by one‐way ANOVA with linear trend analysis to assess the time‐dependent effect (P trend < 0.0001). (N) Analysis of the correlation between mtRC levels and the degree of carcinogenesis. Data are presented as mean ± SEM from three independent experiments. ^*^ *P* < 0.05, ^**^ *P* < 0.01, ^***^ *P* < 0.001, ^****^ *P* < 0.0001.

To explore the function of m^5^C‐modified tsRNAs in BC, we first performed an m^5^C‐specific methylated small RNA immunoprecipitation (RIP) microarray analysis in SV‐HUC‐1, CdCl_2_‐transformed malignant cells (Cd‐SV‐HUC‐1), and T24 cells. Here, 904 m^5^C modified tsRNAs were significantly upregulated (FC ≥ 1.5), whereas seven m^5^C‐tsRNAs were markedly downregulated (FC ≤ 0.667) in Cd‐SV‐HUC‐1 cells relative to SV‐HUC‐1 controls (Figure 1A). Additionally, 779 m^5^C‐tsRNAs were upregulated, and 27 were downregulated compared to SV‐HUC‐1 cells in T24 cells (Figure 1B). Relative to Cd‐SV‐HUC‐1, 190 m^5^C‐tsRNAs increased, and 46 decreased in T24 cells (Figure 1C). Intersection analysis identified five commonly upregulated and only one commonly downregulated m^5^C‐tsRNA across the three comparisons (Figure 1D,E). We further detected the sites of m^5^C modification in tRNA in T24 cells using tRNA bisulfite sequencing and found 33 m^5^C sites in tRNA (Figure S1). The intersection between tRNAs containing the m^5^C modification and six coregulated m^5^C‐modified tsRNAs in the three sets was analyzed. We obtained only one m^5^C methylated tsRNA ‐m^5^C‐tRF3b‐Cys^GCA^‐23 (mtRC) (Figure 1F).

To detect the level of mtRC, we conducted m^5^C IP followed by 3′/5′‐adaptor ligation RT‐PCR assays (Figure 1G). mtRC levels were significantly reduced in Cd‐SV‐HUC‐1 and T24 cells relative to SV‐HUC‐1 cells, with the lowest abundance in T24 cells (Figure 1H). The result was further confirmed by Northern blot assays (Figure 1I). In clinical BC specimens and our previously established multi‐stage bladder cancer tissues [18], mtRC abundance was lower in tumor tissues than in controls (Figure 1J) and progressively declined with prolonged CdCl_2_ exposure and increasing carcinogenic severity (Figure 1K). The level of mtRC was also reduced in the urine specimens from BC patients compared with healthy individuals (Figure 1L). Consistently, urine from the multi‐stage rat bladder cancer [18], showed a stepwise decline in mtRC that inversely correlated with carcinogenic progression (Figure 1M,N), suggesting that mtRC may serve as a biomarker for bladder carcinogenesis.

### 2.2 mtRC Suppresses BC Cell Growth in a Modification‐Dependent Manner

**FIGURE 2:**
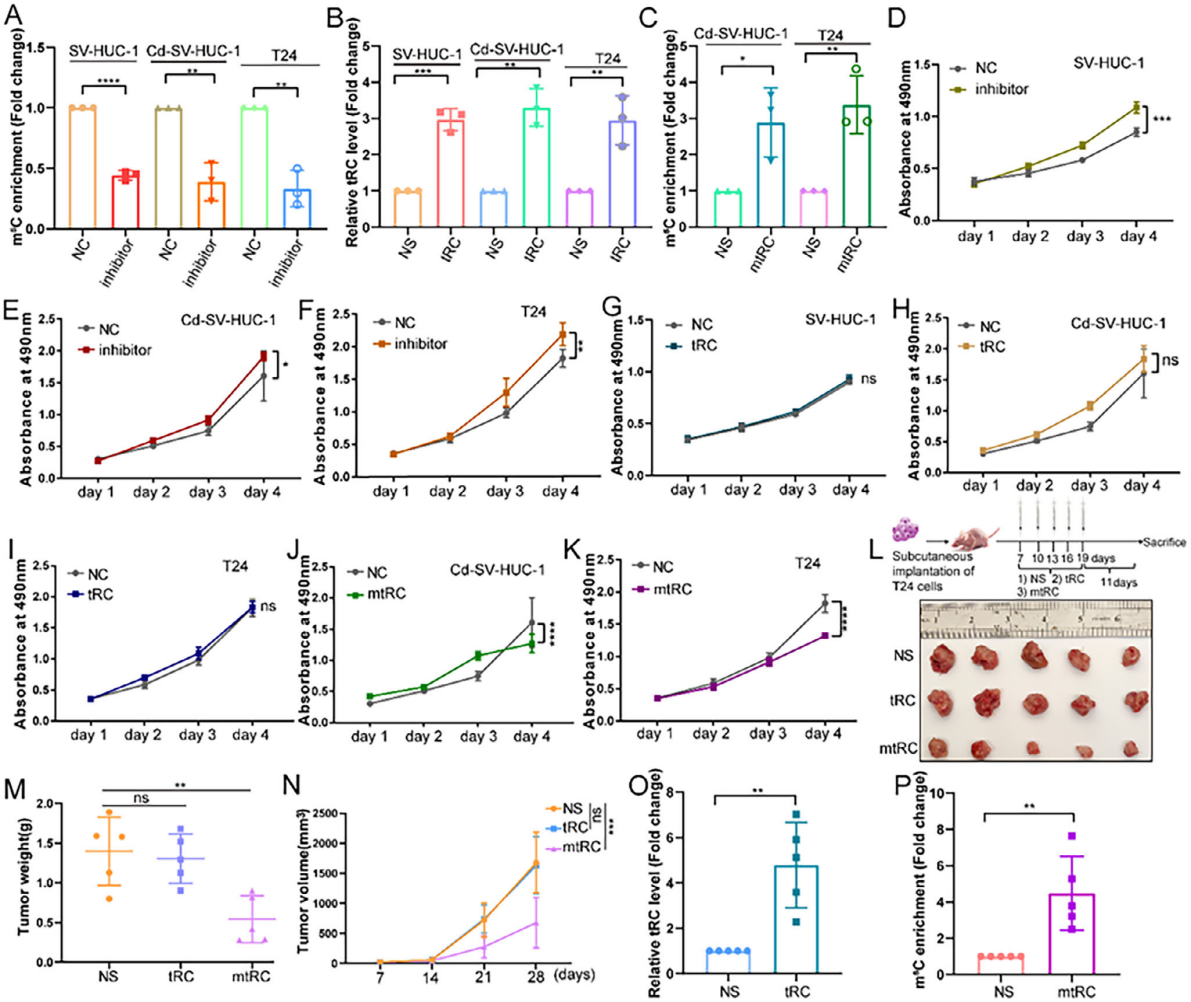
The m^5^C‐tRF3b‐Cys^GCA^‐23 suppresses BC growth. (A) mtRC levels were decreased in SV‐HUC‐1, Cd‐SV‐HUC‐1 and T24 cells after transfected inhibitor. Statistical significance was determined by two‐tailed unpaired t‐test. (B) Overexpression of tRC in SV‐HUC‐1, Cd‐SV‐HUC‐1 and T24 cells. Statistical significance was determined by two‐tailed unpaired t‐test. (C) Overexpression of mtRC in SV‐HUC‐1, Cd‐SV‐HUC‐1 and T24 cells. Statistical significance was determined by two‐tailed unpaired t‐test. (D–F) Cell proliferative capacity was significantly increased by small interfering RNA in SV‐HUC‐1, Cd‐SV‐HUC‐1 and T24 cells. Statistical significance was determined by repeated Measures ANOVA. (G–I) Cell proliferation assay indicated that tRC (without m^5^C modification) overexpression does not affect cell proliferation in SV‐HUC‐1, Cd‐SV‐HUC‐1, and T24 cells. Statistical significance was determined by repeated measures ANOVA. (J, K) Overexpression of mtRC significantly downregulated the ability of proliferation in Cd‐SV‐HUC‐1 and T24 cells. Statistical significance was determined by repeated measures ANOVA. (L–N) Both tumor weight (M) and tumor volume (N) were markedly decreased with mtRC‐agomir treatment compared to the NS antagomir group, whereas tRC overexpression did not influence tumor growth. (Each group *n* = 5). (O) tRC‐agomir treatment significantly increased tRC expression. (P)The mtRC‐agomir treatment considerably increased mtRC expression. Data are presented as mean ± SEM from three independent experiments. ^*^ *P* < 0.05, ^**^ *P* < 0.01, ^***^ *P* < 0.001, ^****^ *P* < 0.0001.

To interrogate mtRC function, we synthesized mtRC inhibitors and mimics, as well as a mimic of the unmodified tRF3b‐Cys^GCA^‐23 (tRC) and introduced them into cells (Figure 2A–C). In MTT assays, mtRC inhibition increased the proliferation of SV‐HUC‐1, Cd‐SV‐HUC‐1, and T24 cells (Figure 2D–F). In contrast, tRC overexpression had no impact on the cell proliferation (Figure 2G–I), whereas mtRC overexpression robustly suppressed proliferation (Figure 2J,K). These results indicate that the biological functions of mtRC depend on m^5^C modification.

We next assessed mtRC activity in vivo using a T24 xenograft model in nude mice. One week after inoculation with T24 cells, tumors received five multisite intratumoral injections every three days with NS agomir, tRC agomir, or mtRC agomir. Compared with the NS agomir group, the mtRC agomir group showed significant reductions in both tumor weight and volume, whereas the tRC agomir group showed no effect on tumor growth (Figure 2L–N). Furthermore, RT‐qPCR results confirmed increased mtRC or tRC abundance in the corresponding groups (Figure 2O,P). Collectively, these data demonstrate a modification‐dependent tumor‐suppressive effect of mtRC in vivo.

### 2.3 NSUN6 Regulates the Abundance of mtRC

**FIGURE 3:**
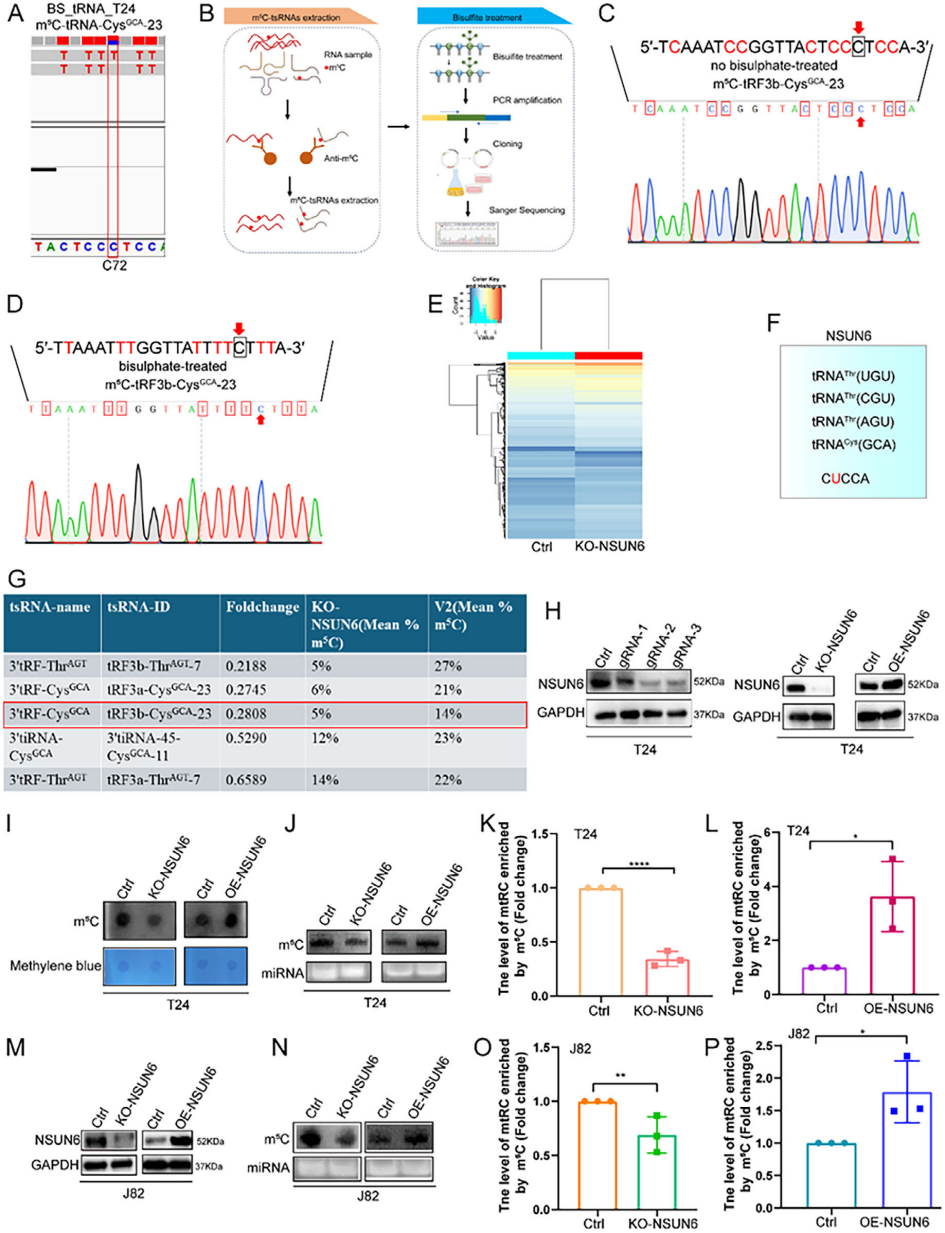
NSUN6 regulates the abundance of mtRC. (A) IGV of tRNA bisulfite sequencing data revealed that the m^5^C methylation site of mtRC is located at C72 (parental tRNA). (B) Flow chart of m^5^C immunoprecipitation combined with bisulfite conversion assay (m^5^C‐BS‐RNA) (C, D) The m^5^C site on mtRC was detected using the m^5^C IP combined with bisulfite conversion assay (BS‐RNA). (E) m^5^C‐specific methylated small RIP microarray analysis in NSUN6‐knockdown Cd‐SV‐HUC‐1 and control cells. (F,G) After the knockdown of NSUN6, the levels of tsRNAs modified by m^5^C, which is mediated by NSUN6, are significantly reduced. (H) The stable cells with knockout NSUN6 were constructed using the CRISPR/Cas9 gene editing technology. NSUN6‐knockout stable Cd‐SV‐HUC‐1 cell lines were established and overexpressed NSUN6 T24 cells. (I,J) Dot blot (G) and Northwestern blot (H) assays revealed that NSUN6 knockdown significantly reduced the m^5^C levels, whereas NSUN6 overexpression enhanced the m^5^C levels in T24 cells. (K,L) The levels of mtRC in NSUN6‐depleted (K) and overexpressed T24 cells (L) were detected using m^5^C IP‐3′/5′ adaptor ligation RT‐PCR. Statistical significance was determined by two‐tailed unpaired t‐test. (M) Construction of knockout NSUN6 and overexpressed NSUN6 stable J82 cells. (N) Northwestern blot assays revealed that NSUN6 knockdown significantly reduced the m^5^C levels, whereas NSUN6 overexpression enhanced the m^5^C levels in J82 cells. (O,P) The levels of mtRC in NSUN6‐depleted (O) and overexpressed J82 cells (P) were detected using m^5^C IP‐3′/5′ adaptor ligation RT‐PCR. Statistical significance was determined by two‐tailed unpaired t‐test. Data are presented as mean ± SEM from three independent experiments. ^*^ *P* < 0.05, ^**^ *P* < 0.01, ^****^ *P* < 0.0001.

Because mtRC derives from tRNA‐Cys (GCA) and carries a site‐specific m^5^C mark, we next sought to identify the methyltransferase responsible for mtRC biogenesis and to validate the modification site. Our tRNA bisulfite sequencing data and Integrative Genomics Viewer (IGV) showed that the methylated cytosine corresponding to mtRC maps to C72 of the parental tRNA (Figure S1 and Figure 3A). We therefore applied an m^5^C IP combined with bisulfite conversion assay (m^5^C‐BS‐RNA) to detect the modification site on mtRC (Figure 3B) and confirmed methylation at the expected position (Figure 3C,D). NSUN6 has been reported to catalyze m^5^C72 on tRNA Cys (GCA) and tRNA Thr (UGU/CGU/AGU) [26, 27].

To determine whether NSUN6 regulates the abundance of mtRC, we performed m^5^C‐specific methylated small RIP microarray analysis in NSUN6‐knockdown Cd‐SV‐HUC‐1 cells and control cells. Four m^5^C‐tsRNAs derived from parental tRNAs harboring C72 m^5^C modification were significantly downregulated upon NSUN6 depletion (Figure 3E–G), including mtRC (Figure 3G). Dot blot and Northwestern blot assays revealed that NSUN6 knockdown reduced global m^5^C levels, whereas NSUN6 overexpression increased global m^5^C levels in T24 cells (Figure 3H–J). Consistently, m^5^C IP‐3′/5′ adaptor ligation RT‐PCR demonstrated that NSUN6 knockdown significantly decreased mtRC abundance, while overexpression increased the mtRC level (Figure 3K,L). Similar results were obtained in J82 cells with NSUN6 knockout or overexpression (Figure 3M–P). To assess whether NSUN6 affects angiogenin (ANG) sensitivity, small RNAs were isolated from NSUN6‐knockdown and control cells and subjected to ANG treatment in vitro. No significant differences in tRF abundance were observed between the two groups after ANG digestion, indicating that NSUN6 does not alter ANG‐mediated tRNA cleavage (Figure S2). Together, these data establish NSUN6 as an upstream determinant of mtRC abundance.

### 2.4 NSUN6 is Downregulated in BC and Inhibits Cell Proliferation

Previous studies have shown that NSUN6 exerts tumor‐suppressive effects in pancreatic and lung cancers [28, 29]. However, its role in BC remains unknown. To evaluate the clinical relevance of NSUN6 in BC, we analyzed TCGA datasets and found that NSUN6 expression was lower in high‐grade than in low‐grade tumors (Figure S3A) and was also reduced in advanced‐stage (III/IV) compared with early‐stage (I/II) BC (Figure S3B). We further examined NSUN6 expressions in BC tissues and in multi‐stage bladder cancer models previously constructed using CdCl_2_ exposure [18]. Immunohistochemistry staining revealed decreased NSUN6 expression in BC tissues compared to cystitis tissues, with the muscle‐invasive bladder cancer (MIBC) group exhibiting the lowest levels (Figure S3C,D). Moreover, NSUN6 abundance declined progressively with increasing CdCl_2_ exposure and tumor malignancy (Figure S3E,F). Consistently, NSUN6 expression levels were markedly decreased in Cd‐SV‐HUC‐1 and BC cells relative to the SV‐HUC‐1 control (Figure S3G), supporting a tumor‐suppressive association.

Functional assays further supported this association. CCK‐8 and EdU assays showed that NSUN6 depletion strongly enhanced the proliferation ability of Cd‐SV‐HUC‐1 and J82 cells (Figure S3H–N), whereas NSUN6 overexpression suppressed T24 cells proliferation (Figure S3O–Q). Moreover, patients exhibiting higher NSUN6 levels exhibited dramatically longer survival times (Figure S3R). Together, these results suggest that NSUN6 acts as a tumor‑suppressive regulator in BC.

### 2.5 mtRC Binds to RBM4 and Enhances RBM4 Protein Stability

**FIGURE 4:**
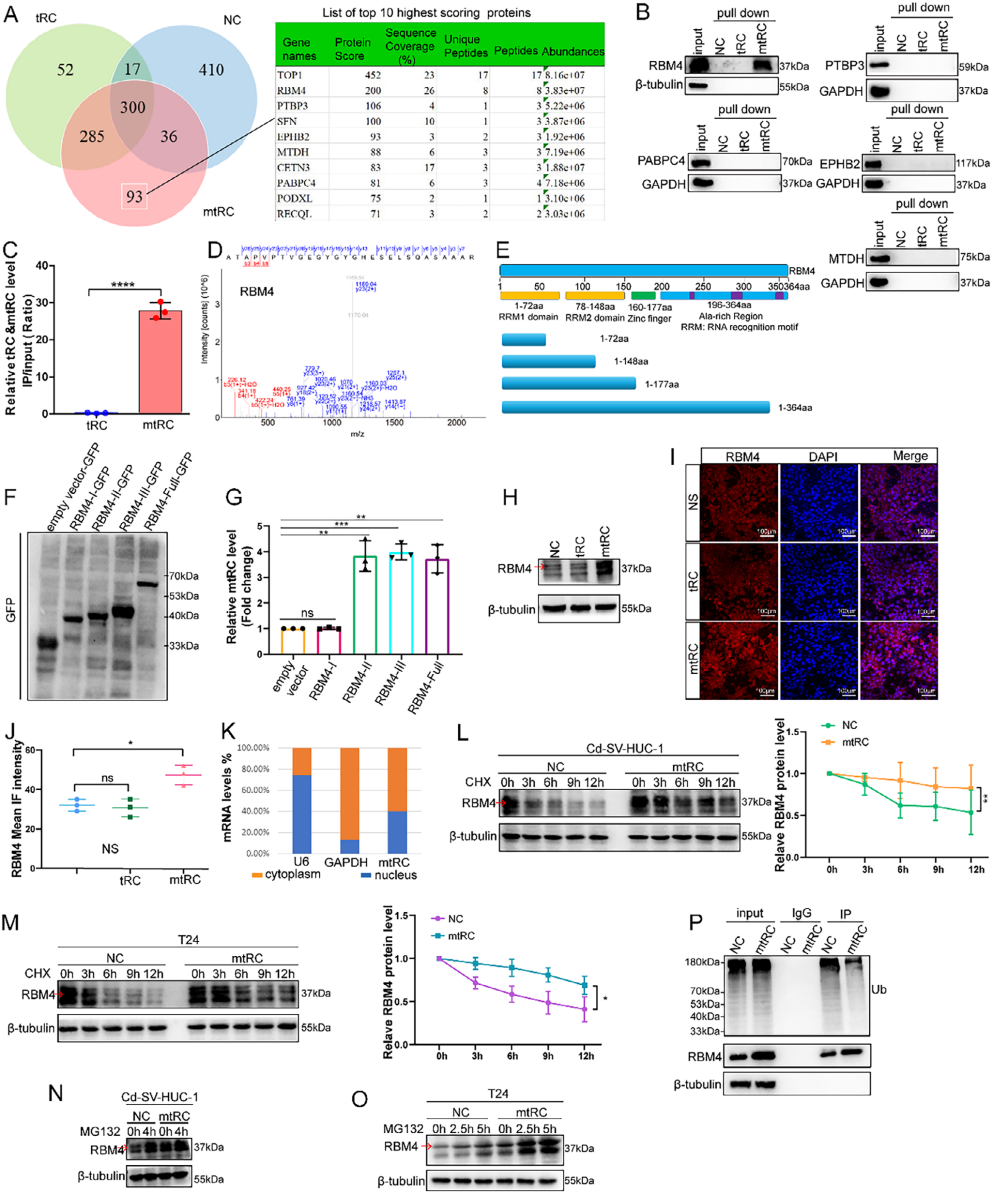
mtRC binds to RBM4 and upregulates its expression. (A) A Venn analysis on the pull‐down proteins using RNA pull‐down assays. (B) RNA pull‐down assays revealed that RBM4 interacted with mtRC. (C) The interaction between RBM4 and mtRC was further validated by RIP assays. Statistical significance was determined by two‐tailed unpaired t‐test. (D) Profile of RBM4 protein. (E) RBM4 protein structure analysis. (F) Vectors carrying GFP‐tagged truncated and full‐length RBM4 were generated. Transfected proteins were verified by WB. (G) RIP assays demonstrated that mtRC interacts with domain II of RBM4. Statistical significance was determined by two‐tailed unpaired t‐test. (H–J) WB and immunofluorescence results demonstrated that mtRC overexpression upregulated the level of RBM4, whereas overexpression of tRC did not affect RBM4 protein levels. Statistical significance was determined by two‐tailed unpaired t‐test. (K) Nucleocytoplasmic separation assay indicated that most mtRC was distributed in the cytoplasm. (L, M) CHX chase results indicated that overexpression of mtRC in T24 and Cd‐SV‐HUC‐1 cells enhanced RBM4 protein stability. Statistical significance was determined by repeated measures ANOVA. (N–P) Deubiquitination assays indicated that mtRC repressed ubiquitin/proteasome‐dependent degradation of RBM4 protein. Data are presented as mean ± SEM from three independent experiments. ^*^ *P* < 0.05, ^**^ *P* < 0.01, ^***^ *P* < 0.001, ^****^ *P* < 0.0001.

To explore the underlying mechanism by which mtRC suppresses bladder tumorigenesis, we synthesized biotin‐conjugated tRC, mtRC, and negative control (NC) probes for pull‐down assays. Intersection analysis identified 93 proteins that bound specifically to mtRC (Figure 4A). We selected the top 10 candidates based on protein score and evaluated their expression in the TCGA database. Expression levels of five genes (TOP1, PODXL, SFN, CETN3, and RECQL) showed no significant difference in BC, whereas the levels of RBM4, PTBP3, EPHB2, MTDH, and PABPC4 exhibited significant differences (Figure S4). To further examine whether the five differentially expressed genes (DEGs) bind to mtRC, we performed RNA pull‐down assays. The results revealed that only RBM4 bound to mtRC (Figure 4B). The interaction between RBM4 and mtRC was further validated by RIP assays (Figure 4C). The profile of the RBM4 protein is indicated in Figure 4D. Analysis of the RBM4 protein structure revealed the presence of RNA‐binding regions (Figure 4E).

To further investigate the specific domain responsible for RBM4 binding to mtRC, we generated GFP‐tagged RBM4 truncation constructs (region I (1‐72aa), region I–II (1‐148aa), region I–III (1‐177aa)) and full‐length RBM4 (region I–IV (1‐364aa)) (Figure 4E and Figure S5A). After expression in 293T cells (Figure 4F), RIP assays revealed that mtRC binds region II of RBM4 (Figure 4G). Western blot (WB) results indicated that mtRC overexpression upregulated the level of RBM4, whereas tRC overexpression did not (Figure 4H). Immunofluorescence experiments further confirmed these results (Figure 4I,J).

The nucleocytoplasmic separation assay indicated that most mtRC is predominantly cytoplasmic (Figure 4K). Cycloheximide (CHX) chase experiments further demonstrated that mtRC prolongs RBM4 protein half‐life in both T24 and Cd‐SV‐HUC‐1 cells (Figure 4L,M). Ubiquitination assays indicated that mtRC decreases ubiquitin/proteasome‐dependent turnover of RBM4 (Figure 4N–P). According to UniProt annotations, ubiquitination sites of RBM4 are mainly distributed in Region I and Region II, with five ubiquitination sites located within Region II (Figure S5B,C). Collectively, these data show that mtRC binds to RBM4 and enhances RBM4 protein stability by blocking its ubiquitination.

### 2.6 RBM4 is Expressed at a Low Level in BC and Inhibits Cell Proliferation

**FIGURE 5:**
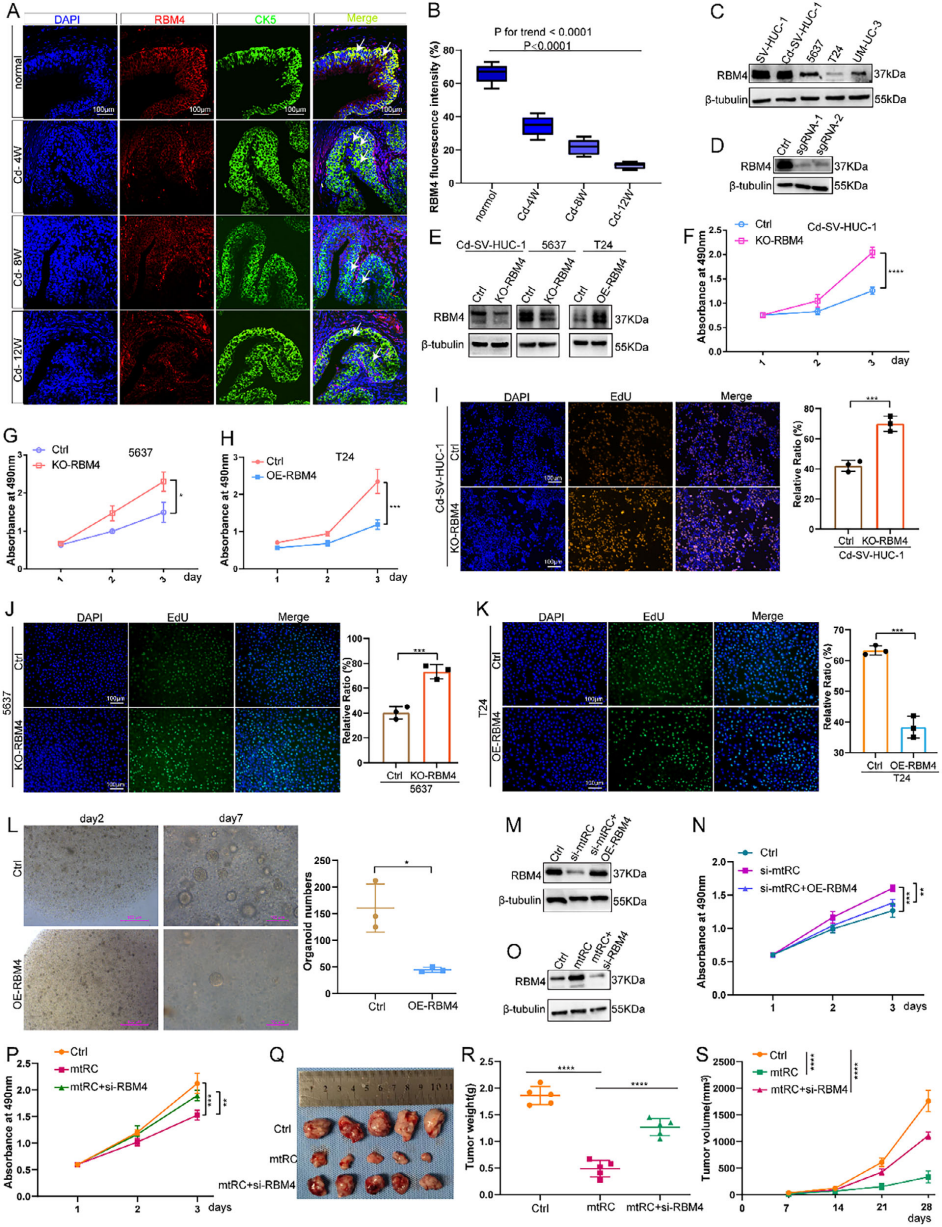
RBM4 is expressed at a low level in BC and inhibits cell proliferation. (A,B) Immunofluorescence indicated that the expression level of RBM4 was gradually downregulated with the increasing extent of carcinogenesis. Data are presented as box plots showing the median and quartiles. Statistical significance was determined by one‐way ANOVA with linear trend analysis to assess the time‐dependent effect (P trend < 0.0001). (C) the RBM4 protein levels were examined in Cd‐SV‐HUC‐1 and BC cells using WB. (D) The stable cells with knockout RBM4 were constructed using the CRISPR/Cas9 gene editing technology. (E) Construction of knockout RBM4 stable cells and overexpressed RBM4 cells. (F,G) CCK‐8 assays revealed that RBM4 depletion strongly upregulated Cd‐SV‐HUC‐1 and 5637 cell growth. Statistical significance was determined by repeated measures ANOVA. (H) CCK‐8 assays revealed that RBM4 overexpression downregulated the proliferation ability of T24 cells. Statistical significance was determined by repeated measures ANOVA. (I,J) EdU assays also revealed that RBM4 knockdown enhances cell proliferation. Statistical significance was determined by two‐tailed unpaired t‐test. (K) EdU assays also revealed that RBM4 overexpression suppresses the proliferation ability of cells. Statistical significance was determined by two‐tailed unpaired t‐test. (L) BC patient‐derived organoid experimental results also indicated that overexpression of RBM4 significantly inhibits BC organoids growth. (M,N) CCK‐8 assays demonstrated that RBM4 overexpression suppressed cell proliferative capability in mtRC‐downregulated Cd‐SV‐HUC‐1 cells. Statistical significance was determined by repeated measures ANOVA. (O,P) A decrease of RBM4 significantly rescued the proliferation in mtRC‐overexpressing Cd‐SV‐HUC‐1 cells. Statistical significance was determined by repeated measures ANOVA. (Q–S) mtRC overexpression markedly inhibited tumor growth, whereas RBM4 silencing partially reversed this inhibitory effect, as reflected by increased tumor weight (R) and tumor volume (S) compared with the mtRC overexpression group (Each group *n* = 5). Data are presented as mean ± SEM. ^*^ *P* < 0.05, ^**^ *P* < 0.01, ^***^ *P* < 0.001, ^****^ *P* < 0.0001.

To interrogate RBM4's function in BC, we examined its expression level in multi‐stage bladder carcinogenesis model [18] and found that RBM4 expression in CK5‐positive urothelial epithelial cells was progressively reduced as the duration of CdCl_2_ treatment increased (Figure 5A,B), indicating a gradual loss of RBM4 during bladder carcinogenesis. RBM4 protein was also decreased in both Cd‐SV‐HUC‐1 and BC cells relative to SV‐HUC‐1 control (Figure 5C). Subsequently, stable RBM4 knockdown cells and overexpression cell lines were established (Figure 5D,E). CCK‐8 and EdU experiments showed that RBM4 depletion strongly enhanced the proliferation of Cd‐SV‐HUC‐1 and 5637 cells (Figure 5F,G,I,J). Conversely, RBM4 overexpression suppressed the proliferation ability of T24 cells (Figure 5H,K). RBM4 overexpression also inhibited the growth of patient‐derived BC organoids (Figure 5L), supporting a tumor‐suppressive role.

To determine whether RBM4 mediates the effects of mtRC on cell proliferation, we overexpressed RBM4 in mtRC‐depleted Cd‐SV‐HUC‐1 cells (Figure 5M). Restoration of RBM4 overexpression blunted the increased proliferation caused by mtRC loss (Figure 5N). Conversely, RBM4 attenuated mtRC‐induced proliferation suppression in T24 cells (Figure 5O,P). We further validated this relationship in vivo using a subcutaneous xenograft model. One week after subcutaneous implantation of T24 cells into nude mice, the animals were randomized into three groups: negative control (NC), mtRC overexpression, and mtRC overexpression combined with RBM4 knockdown via siRNA. Tumor volumes were monitored weekly, and tumors were harvested and weighed four weeks after implantation. Consistent with our in vitro findings, mtRC overexpression markedly inhibited tumor growth, whereas RBM4 silencing partially reversed this inhibitory effect, as reflected by increased tumor volume and tumor weight compared with the mtRC overexpression group (Figure 5Q–S). The data indicate that mtRC inhibits BC cell growth largely through RBM4.

### 2.7 mtRC Suppresses the Glycolytic Capacity of BC Cells by Regulating RBM4

**FIGURE 6:**
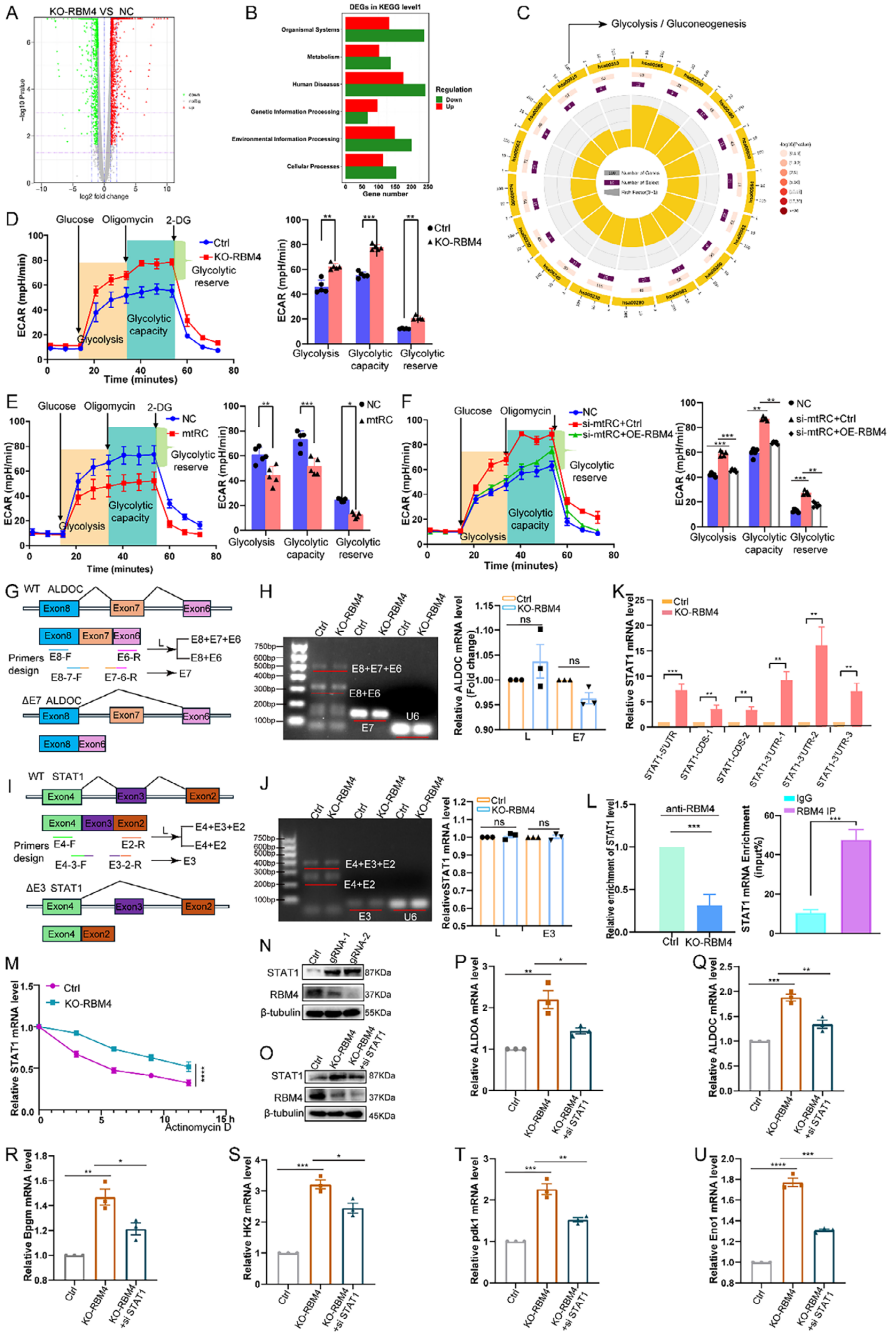
RBM4 suppresses glycolytic capacity in BC cells. (A) DEGs were detected by RNA‐seq analysis in Cd‐SV‐HUC‐1 cells with RBM4 knockdown compared to control cells using RNA‐seq. (B) DEGs were assessed for pathway enrichment in metabolism using the KEGG pathway analysis. (C) Pathway analysis of DEGs involved in the glycolytic pathway. (D) ECAR results revealed that RBM4 knockdown accelerated glycolysis in Cd‐SV‐HUC‐1 cells. Statistical significance was determined by two‐tailed unpaired t‐test. (E) ECAR assays indicated that overexpression of mtRC inhibited glycolysis in T24 cells. Statistical significance was determined by two‐tailed unpaired t‐test. (F) ECAR experiment indicated that overexpression of RBM4 rescued glycolysis in mtRC knockdown Cd‐SV‐HUC‐1 cells. Statistical significance was determined by two‐tailed unpaired t‐test. (G) Schematic illustration of RT‐qPCR primer design targeting two ALDOC isoforms (wild‐type and exon 7–skipped). (H) RBM4 knockdown did not alter the exon inclusion pattern of ALDOC. Statistical significance was determined by two‐tailed unpaired t‐test. (I) Schematic illustration of RT‐qPCR primer design targeting two STAT1 isoforms (wild‐type and exon 3–skipped). (J) RBM4 knockdown did not alter the exon inclusion pattern of STAT1. Statistical significance was determined by two‐tailed unpaired t‐test. (K) RT–qPCR showed that RBM4 knockdown upregulated STAT1 mRNA levels. Statistical significance was determined by two‐tailed unpaired t‐test. (L) RNA immunoprecipitation (RIP) assays showed that RBM4 bound to STAT1 mRNA, while STAT1 mRNA enrichment was substantially reduced upon RBM4 silencing. Statistical significance was determined by two‐tailed unpaired t‐test. (M) RBM4 knockdown increased STAT1 mRNA stability. Statistical significance was determined by repeated measures ANOVA. (N) RBM4 depletion resulted in elevated STAT1 protein expression. (O–U) STAT1 knockdown in RBM4‐silenced cells reduced the expression of multiple glycolytic genes. Statistical significance was determined by two‐tailed unpaired t‐test. Data are presented as mean ± SEM from three independent experiments. ^*^ *P* < 0.05, ^**^ *P* < 0.01, ^***^ *P* < 0.001, ^****^ *P* < 0.0001.

To interrogate the regulation mechanism of RBM4, RNA‐seq analysis was performed on RBM4‐knockdown and control Cd‐SV‐HUC‐1 cells. A total of 1,559 genes were upregulated, and 1,535 genes were downregulated (FC ≥ 2, *P* < 0.05) upon RBM4 depletion (Figure 6A). Subsequently, these DEGs were assessed for metabolic pathway enrichment using the KEGG pathway analysis, which revealed that the DEGs were mainly involved in the glycolytic pathway (Figure 6B,C).

To further evaluate the impact of RBM4 on glycolysis, we analyzed the ECAR of RBM4‐knockdown Cd‐SV‐HUC‐1 and 5637 cells, revealing that the knockdown of RBM4 accelerated glycolysis (Figure 6D; Figure S6A,B). mtRC overexpression in T24 and 5637 cells inhibited glycolysis (Figure 6E; Figure S6C,D). Importantly, RBM4 overexpression rescued the glycolytic elevation induced by mtRC knockdown in Cd‐SV‐HUC‐1 and 5637 cells (Figure 6F; Figure S6E,F). In line with RNA‐seq (Figure S7A), RT‐qPCR results confirmed that RBM4 depletion upregulated multiple glycolytic genes (Figure S7B–F).

Given that RBM4 is a well‐characterized splicing factor, we first investigated whether RBM4 regulates glycolysis through alternative splicing of glycolytic genes. Comprehensive splicing analysis revealed that among glycolysis‐related genes, only ALDOC exhibited a differential exon‐skipping event (Figure S7G–K). We designed RT‐qPCR primers specifically targeting two isoforms of ALDOC (Figure 6G; Table S1). However, the data showed that RBM4 knockdown did not alter the exon inclusion pattern of ALDOC (Figure 6H), indicating that RBM4‐dependent alternative splicing is unlikely to account for the observed glycolytic changes.

Previous reports indicate that RBM4 modulates glycolysis by targeting STAT1 [30], a transcription factor that has been documented to regulate the expression of multiple glycolytic genes [31]. We next investigated whether RBM4 regulates glycolysis through STAT1 signaling. Based on our RNA‐seq analysis, we first assessed whether RBM4 regulates STAT1 through alternative splicing. Although the RNA‐seq data suggested that STAT1 exon‐skipping events were markedly altered upon RBM4 knockdown (Figure S7K), the results of RT‐qPCR showed no significant change in the corresponding splicing event after RBM4 silencing (Figure 6I,J; Table S3) Notably, our RNA‐seq data showed that RBM4 knockdown upregulated STAT1 mRNA levels (Figure S7A), which was further confirmed by RT‐qPCR (Figure 6K).

To elucidate how RBM4 regulates STAT1 expression, we performed RNA immunoprecipitation (RIP) assays and found that RBM4 bound to STAT1 mRNA, while STAT1 mRNA enrichment was substantially reduced upon RBM4 silencing (Figure 6L). Consistently, mRNA stability assays indicated that RBM4 knockdown increased STAT1 mRNA stability, leading to elevated STAT1 expression (Figure 6M). Furthermore, we found that RBM4 depletion resulted in elevated STAT1 protein expression (Figure 6N). STAT1 knockdown in RBM4‐silenced cells reduced the expression of multiple glycolytic genes, including ALDOA, ALDOC, BPGM, HK2, PDK1, and ENO1 (Figure 6O–U), indicating that STAT1 is a key mediator of the glycolytic gene upregulation induced by RBM4 loss. Collectively, these results suggest that RBM4 suppresses glycolytic capacity, at least in part, by destabilizing STAT1 mRNA, thereby attenuating STAT1‐dependent transcription of glycolytic genes.

### 2.8 Effects of H3K18 Lactylation Inhibition by mtRC on BC Cell Proliferation

**FIGURE 7:**
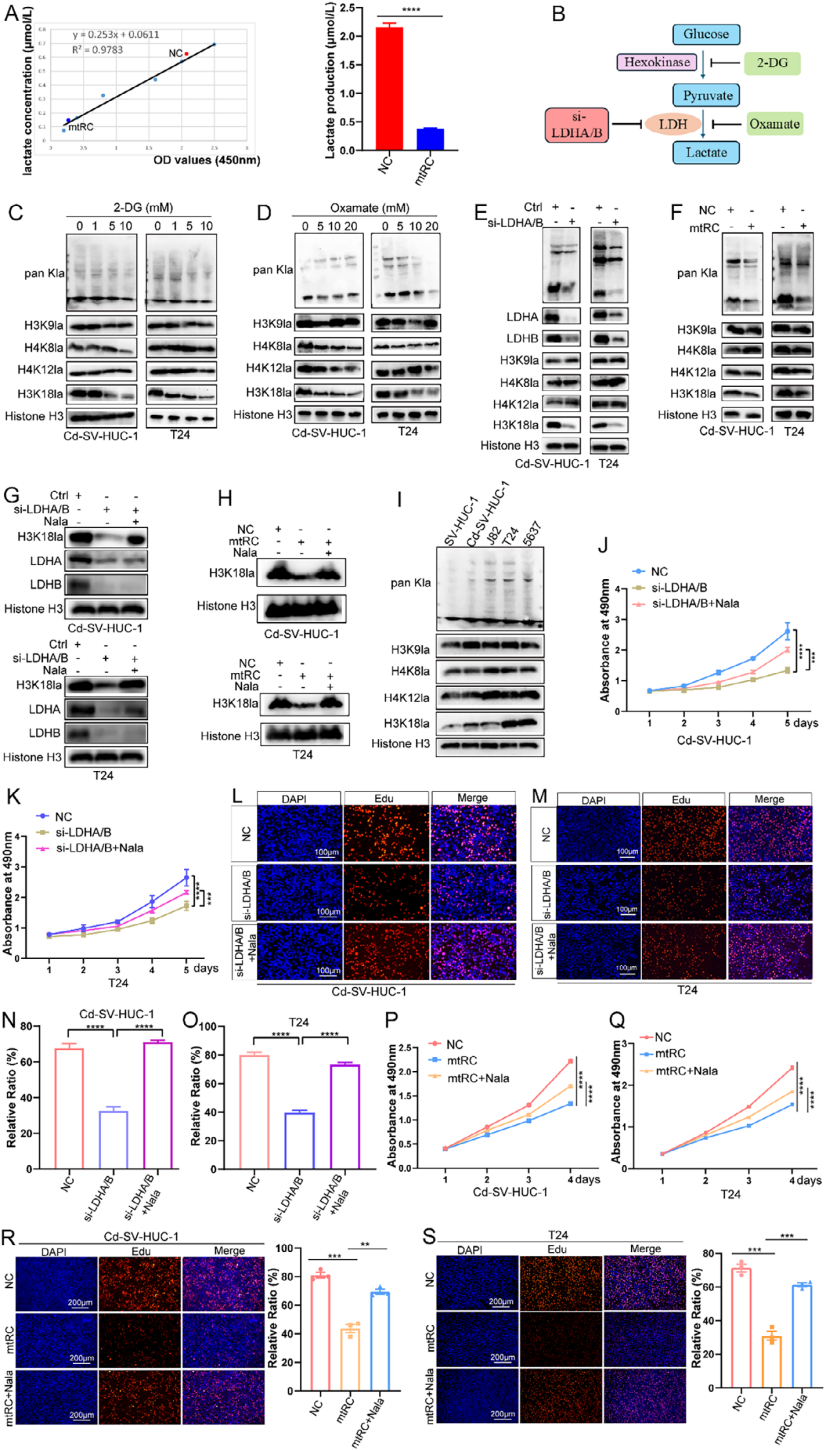
Effects of histone lactylation inhibition by mtRC on BC cells proliferation. (A) the total lactate produced was measured in T24 cells with overexpressed mtRC. (B) Schematic of glycolysis, indicating the strategies used in this study to inhibit lactate production and histone lactylation. (C,D) Cells were treated with varying concentrations of the glycolysis inhibitors 2‐DG or oxamate for 24 h to assess their effects on pan‐Kla and histone lactylation. (E) the levels of pan‐Kla and histone lactylation upon LDH depletion were assessed using Western blotting. (F) mtRC reduced intracellular levels of pan‐Kla and H3K18la. (G) Lactate addition restored H3K18la in LDHA/LDHB‐knockdown SV‐HUC‐1 and T24 cells. (H) Lactate addition restored H3K18la in mtRC‐overexpressing cells. (I) The levels of pan‐Kla and histone lactylation were measured in SV‐HUC‐1 and BC cell lines. (J–O) CCK‐8 (J,K) and EdU (L–O) experiments showed that LDHA/LDHB knockdown strongly reduced the proliferation of Cd‐SV‐HUC‐1 and T24 cells, whereas sodium lactate partially restored growth. (J,K) Statistical significance was determined by repeated measures ANOVA. (N, O) Statistical significance was determined by two‐tailed unpaired t‐test. (P–S) CCK‐8 (P,Q) and EdU (R,S) assays showed that mtRC overexpression significantly suppressed cell proliferation, and this inhibitory effect was partially rescued by sodium lactate treatment. (P,Q) Statistical significance was determined by repeated measures ANOVA. (R,S) Statistical significance was determined by two‐tailed unpaired t‐test. Data are presented as mean ± SEM. ^**^ *P* < 0.01, ^***^ *P* < 0.001, ^***^ *P* < 0.0001.

Because mtRC suppresses glycolysis, we asked whether it also impacts lactate production and histone lactylation. mtRC overexpression significantly reduced lactate levels in T24 cells (Figure 7A). Given that lactate can modify nuclear histones and influence gene expression [23], we examined global protein lactylation and histone H3K18 lactylation (H3K18la). Pharmacological inhibition of glycolysis with 2‐deoxy‐D‐glucose (2‐DG) or oxamate reduced glycolysis and H3K18la levels in a dose‐dependent manner (Figure 7B–D), and knockdown of lactate dehydrogenase A (LDHA) and lactate dehydrogenase B (LDHB) similarly decreased both marks (Figure 7E). Notably, mtRC overexpression markedly reduced global lactylation and histone lactylation levels (Figure 7F). To test whether the decrease in H3K18la resulted from reduced lactate availability, we supplemented cells with exogenous sodium lactate. Lactate addition restored H3K18la in LDHA/LDHB‐knockdown cells and similarly rescued H3K18la in mtRC‐overexpressing cells (Figure 7G,H). Notably, both global lactylation and H3K18 lactylation levels were significantly upregulated in BC cell lines relative to SV‐HUC‐1 cells (Figure 7I).

To explore the role of histone lactylation in BC, endogenous LDHA and LDHB were knocked out in cells, which were then supplemented with sodium lactate to perform rescue assays. CCK‐8 and EdU experiments indicated that LDHA and LDHB depletion strongly reduced the proliferation of Cd‐SV‐HUC‐1 and T24 cells, whereas sodium lactate restored the inhibited tumor cell growth (Figure 7J–O). Similarly, mtRC overexpression significantly suppressed cell proliferation, and this inhibitory effect was partially rescued by sodium lactate treatment, as assessed by CCK‐8 and EdU assays (Figure 7P–S). Collectively, these data indicate that mtRC reduces histone H3K18 lactylation by limiting lactate production, and that H3K18la promotes BC cell proliferation.

### 2.9 H3K18 Lactylation Enhances Transcription of Oncogenic IL1RAP and VASH2 in BC

**FIGURE 8:**
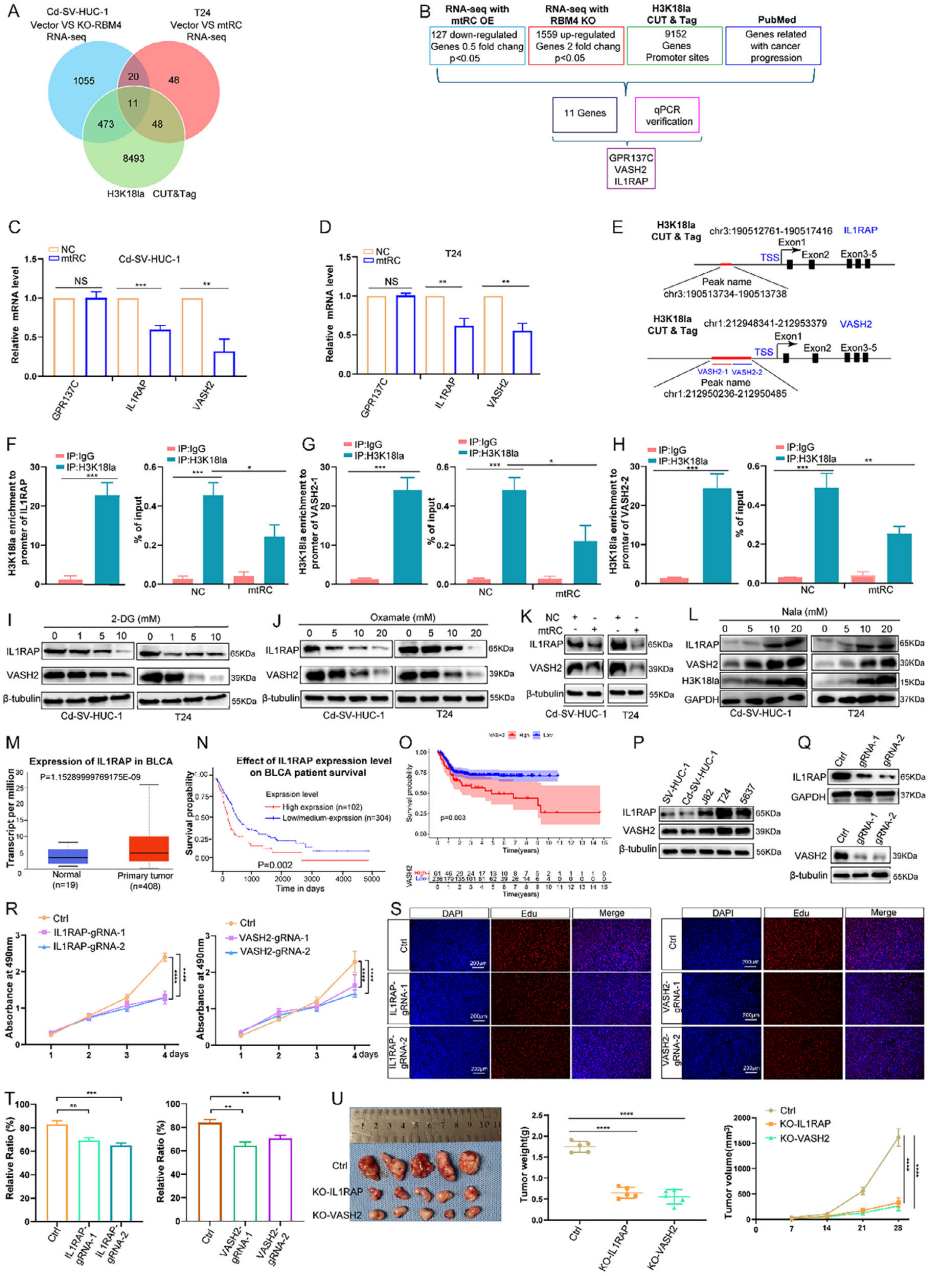
IL1RAP and VASH2 are targets of H3K18 lactylation and function as oncogenes in BC. (A) An intersection analysis was performed among the following datasets: ChIP‐seq data, CUT&Tag results, downregulated genes after mtRC overexpression, and upregulated genes following RBM4 knockdown. (B) Flow diagram illustrating the identification of the downstream regulatory target of H3K18la. (C,D) The mRNA levels of GPR137C, IL1RAP, and VASH2 were assessed in mtRC overexpression cells. Statistical significance was determined by two‐tailed unpaired t‐test. (E) Schematic of H3K18la peaks at the IL1RAP, and VASH2 promoter regions. (F–H) The enrichment of H3K18la at the IL1RAP and VASH2 promoter regions was assessed by ChIP‐qPCR in overexpressing mtRC and control cells. Statistical significance was determined by two‐tailed unpaired t‐test. (I,J) IL1RAP and VASH2 protein expressions were measured by immunoblotting in cells exposed to varying doses of 2‐DG or oxamate. (K) IL1RAP and VASH2 protein expressions were assessed after mtRC overexpression. (L) Sodium lactate supplementation increased H3K18la levels and concomitantly upregulated IL1RAP and VASH2 expression. (M) The IL1RAP levels in TCGA data. (N) Higher IL1RAP levels correlate with worse survival. (O) Elevated VASH2 expression was significantly associated with poorer patient outcomes compared with low VASH2 expression. (P) The protein expression levels of IL1RAP and VASH2 were detected in BC cell lines and control cells. (Q) IL1RAP‐deficient and VASH2‐deficient T24 lines were generated. (R) Cell viability of IL1RAP‐deficient and VASH2‐deficient T24 cells was assessed using the CCK‐8 assay. Statistical significance was determined by repeated measures ANOVA. (S, T) Assessment of proliferation in IL1RAP‐deficient and VASH2‐deficient T24 cells via EdU assay. Statistical significance was determined by two‐tailed unpaired t‐test. (U) Both tumor weight and volume were significantly reduced in the IL1RAP‐ and VASH2‐knockdown groups (Each group *n* = 5) compared with the control group (*n* = 5). Data are presented as mean ± SEM from three independent experiments. ^*^ *P* < 0.05, ^**^ *P* < 0.01, ^***^ *P* < 0.001, ^****^ *P* < 0.0001.

To identify transcriptional programs controlled by H3K18la downstream of mtRC, we performed RNA‐seq in T24 cells with mtRC overexpression and control cells and identified 127 genes significantly downregulated upon mtRC overexpression (Figure 8A,B). We then obtained publicly available ChIP‐seq datasets generated in T24 cells using a ChIP‐grade antibody against H3K18la, as previously described by Bo Xie et al. [24]. By integrating the H3K18la ChIP‐seq data with CUT&Tag results, the list of genes downregulated upon mtRC overexpression, the genes upregulated following RBM4 knockdown, and information retrieved from the PubMed database, we identified GPR137C, IL1RAP, and VASH2 as candidate genes (Figure 8A,B). We next examined the mRNA expression of the candidate targets in mtRC‐overexpressing cells and found that IL1RAP and VASH2 were significantly regulated (Figure 8C,D). Based on Bo Xie's CUT&Tag sequencing data, we designed specific primers targeting distinct regions of the IL1RAP and VASH2 promoters (Figure 8E). Subsequent ChIP analysis demonstrated enrichment of H3K18la at these promoters. Of note, the interaction between H3K18la and the IL1RAP and VASH2 promoters was diminished upon mtRC overexpression (Figure 8F–H; Figure S8A–C), which further supports a transcriptional regulatory mechanism associated with H3K18 lactylation in BC.

We next assessed IL1RAP and VASH2 protein expression levels using Western blot analysis. Glycolysis inhibitors reduced IL1RAP and VASH2 abundance, and mtRC overexpression similarly suppressed the expression of IL1RAP and VASH2 (Figure 8I–K). Notably, sodium lactate supplementation increased H3K18la levels and concomitantly upregulated IL1RAP and VASH2 expression, supporting a lactate/H3K18la‐dependent regulation of these targets (Figure 8L). Analysis of public datasets (TCGA and Kaplan‐Meier Plotter) indicated that IL1RAP is upregulated in BC tissues and higher IL1RAP levels are associated with BC progression and poorer outcomes (Figure 8M,N). RNA sequencing data from 297 MIBC samples with prognostic information were retrieved from three GEO datasets (GSE13507, GSE31684, GSE32894). Prognostic analysis revealed that elevated VASH2 expression was significantly associated with poorer patient outcomes compared with low VASH2 expression (Figure 8O). We further examined the protein expression levels of IL1RAP and VASH2 in BC cell lines. Compared to SV‐HUC‐1, both proteins were found to be upregulated in BC cells (Figure 8P). To investigate their function, we generated T24 and 5637 cell lines with IL1RAP or VASH2 knockdown, respectively (Figure 8Q; Figure S8D,E). CCK8 and EdU assays showed that the knockdown of IL1RAP or VASH2 significantly suppressed cellular proliferation (Figure 8R–T; Figure S8F–K). To further evaluate the effects in vivo, T24 cells with IL1RAP or VASH2 knockdown were subcutaneously implanted into nude mice. Tumor volumes were measured weekly, and tumors were excised and weighed after 4 weeks of growth. Both tumor weight and volume were significantly reduced in the IL1RAP‐ and VASH2‐knockdown groups compared with the control group, indicating that IL1RAP and VASH2 contribute to bladder cancer tumorigenesis in vivo (Figure 8U). Collectively, these findings identify IL1RAP and VASH2 as oncogenic effectors whose transcription is closely associated with H3K18 lactylation in BC.

## 3 Discussion

tsRNAs are increasingly implicated in tumorigenesis and have emerged as promising noninvasive biomarkers across multiple cancer types. In line with previous reports in breast [32] and gastric cancers [33] where specific tRFs show diagnostic or prognostic value [34], we found that mtRC is significantly reduced in BC tissues and urine and inversely correlates with carcinogenesis progression. These findings suggest that mtRC may represent a candidate biomarker for BC, pending further clinical validation.

NSUN6 catalyzes m^5^C modification at C72 of specific tRNAs [26] and requires an intact tRNA substrate, including the CCA end, the cytosine residue at position 72 (C72), the discriminator base U73, and the primary sequence and tertiary structure [22]. Given the stringent structural requirements, mtRC is likely derived from the cleavage of NSUN6‐methylated parental tRNA and inherits the m^5^C methylation at C72 rather than acquiring it post‐cleavage. Our experimental results show that NSUN6 depletion reduces mtRC abundance without affecting angiogenin‐dependent cleavage, indicating that NSUN6 primarily regulates mtRC levels by controlling the pool of methylated precursor tRNA. Although the nuclease responsible for mtRC generation remains unknown, these findings suggest that mtRC biogenesis likely involves alternative processing pathways independent of angiogenin. Identification of the responsible nuclease(s) and potential tsRNA demethylation mechanisms will be important directions for future investigation. NSUN6 has been reported to exert tumor‐suppressive activity in other malignancies, including pancreatic cancer [29], and colon adenocarcinoma [35]. Here, we extend these findings to BC, demonstrating that NSUN6 acts as an upstream regulator of mtRC biogenesis and functions as a tumor suppressor.

RBM4 is a multifunctional RNA‐binding protein implicated in RNA splicing, stability, and translational regulation [36], with tumor‐suppressive roles reported in several malignancies [37, 38, 39, 40]. We demonstrate that RBM4 is progressively downregulated during BC progression and restrains BC cell proliferation. Mechanistically, mtRC directly binds RBM4 and protects it from ubiquitin/proteasome‐dependent degradation, thereby stabilizing RBM4 protein levels. This defines a noncanonical mechanism by which a chemically modified tsRNA regulates protein stability rather than acting through miRNA‐like base pairing.

RBM4 has been implicated in metabolic regulation, with RBM4 depletion enhancing glycolytic activity and RBM4 overexpression suppressing glycolysis [30]. In line with these findings, we observed that RBM4 depletion in BC cells enriched glycolysis‐related transcriptional programs and increased ECAR, indicating enhanced glycolytic capacity. Because elevated glycolysis supports anabolic growth and represents a hallmark of tumorigenesis [41, 42, 43], RBM4‐mediated metabolic restraint may represent an important layer of tumor suppression.

Although several noncoding RNAs, including tRFs [44] and lncRNAs [45, 46, 47] have been reported to regulate glycolysis in cancer, the role of chemically modified tsRNAs in this process has remained largely unexplored. Here, we demonstrate that the m^5^C‐modified tsRNA mtRC suppresses glycolysis in BC cells, and that RBM4 depletion reverses this effect, supporting a model in which an epitranscriptomically modified tsRNA restrains tumor progression by stabilizing RBM4.

Histone lactylation has emerged as a metabolically driven epigenetic modification that contributes to malignant phenotypes across cancer types [23, 24, 25, 48]. Consistent with these studies, we observed elevated H3K18la in BC models and found that this mark promotes BC cell proliferation. By limiting glycolysis and lactate availability, mtRC indirectly constrains H3K18la and attenuates transcription of oncogenic targets such as IL1RAP and VASH2. Although our integrative RNA‐seq, ChIP, and functional knockdown assays establish a strong correlation between H3K18la enrichment and transcription of these targets, direct promoter‐level causal regulation remains to be proven. Future studies involving luciferase reporter assays and promoter mutagenesis will be necessary to confirm this mechanistic link.

Collectively, our study delineates an mtRC–RBM4–glycolysis–H3K18la–IL1RAP/VASH2 regulatory axis that links tsRNA epitranscriptomic modification to metabolic–epigenetic transcriptional control. This mechanism expands the functional repertoire of tsRNAs beyond classical RNA interference and highlights how RNA modifications can impose durable metabolic and chromatin changes in cancer.

Several limitations warrant consideration. First, tumor growth and metabolic regulation were evaluated using a subcutaneous xenograft model, which does not fully recapitulate the orthotopic bladder microenvironment. Second, the E3 ubiquitin ligase(s) responsible for RBM4 turnover remains to be identified. Third, given the limited sample size (12 BC patients and 12 controls) in this study, larger multi‐center cohorts are warranted to confirm the diagnostic utility of urinary mtRC. Finally, promoter‐level validation and overexpression rescue experiments for downstream targets (IL1RAP/VASH2) will further refine the proposed regulatory hierarchy.

**FIGURE 9:**
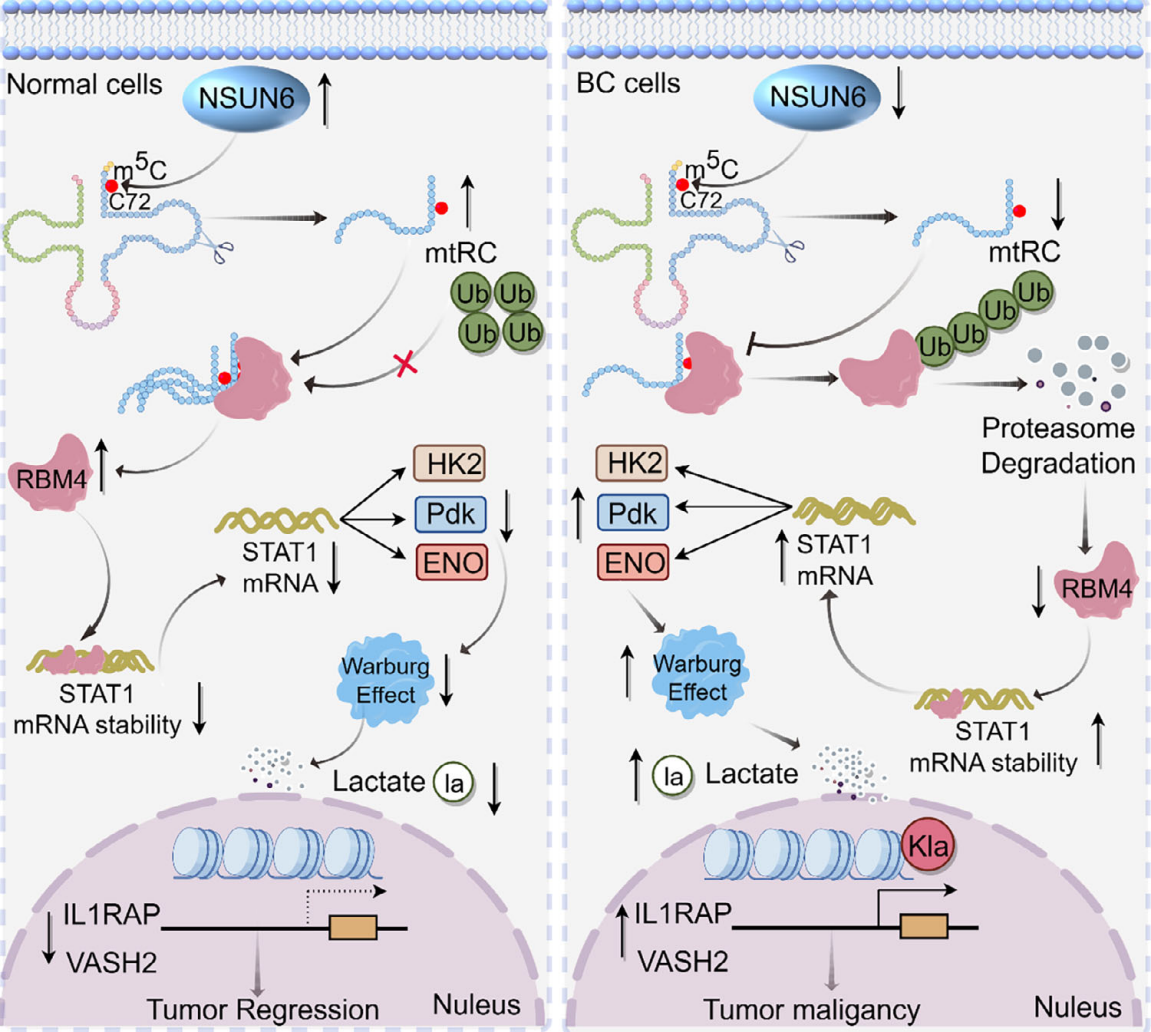
Graphical abstract: Model depicting that mtRC, an NSUN6‐dependent m^5^C‐modified tsRNA, binds and stabilizes RBM4. RBM4 in turn destabilizes STAT1 mRNA, suppressing glycolysis and reducing lactate production. Decreased lactate leads to lower H3K18 lactylation, which dampens the transcription of oncogenic targets IL1RAP and VASH2, ultimately restraining bladder cancer progression.

In summary, we identify mtRC as an NSUN6‐dependent m^5^C‐modified tsRNA that suppresses BC progression by stabilizing RBM4, restraining glycolysis, and limiting oncogenic transcription promoted by histone lactylation (Figure 9). These findings establish a regulatory axis linking epitranscriptomic control to chromatin regulation, with implications for BC diagnosis and therapy.

## 4 Experimental Section

### 4.1 Antibodies

5‐methylcytosine (5‐mC) (Abcam; ab10805, RRID: AB_442823), DIG (Roche, #11093274910, RRID: AB_514497), GFP (Santa Cruz Biotechnology; sc‐9996, RRID: AB_627695), RBM4 antibody (Proteintech; 11614‐1‐AP, RRID: AB_2176095), RBM4 (Proteintech, # 60292‐1‐Ig, RRID: AB_2881407), IgG (Proteintech; 30000‐0‐AP, RRID: AB_2819035), ubiquitin (Cell signaling Technology; 3936S, RRID:AB_331292), PTBP3 (Proteintech, #14027‐1‐AP, RRID: AB_2182321), PABPC4 (Proteintech, #14960‐1‐AP, RRID: AB_2156897), β‐tubulin (Proteintech, #10094‐1‐AP, RRID: AB_2210695), GAPDH (Proteintech, # 10494‐1‐AP, RRID:AB_2263076), CK5 (Abcam, # ab52635, RRID: AB_869890), L‐Lactyl Lysine (PTM BIO, # PTM‐1401RM, RRID:AB_2942013), Lactyl‐Histone H3 (Lys18) (PTM BIO # PTM‐1406RM, RRID:AB_2909438), L‐Lactyl‐Histone H3 (Lys9) (PTM BIO, PTM‐1419RM, RRID:AB_3076695), L‐Lactyl‐Histone H4 (Lys8) (PTM BIO, PTM‐1415RM, RRID:AB_3101829); L‐Lactyl‐Histone H4 (Lys12) (PTM BIO, PTM‐1411RM, RRID: AB_2941896), LDHA (Proteintech # 19987‐1‐AP, RRID: AB_10 646 429), LDHB (Proteintech, # 14824‐1‐AP, RRID: AB_2 134 953).

### 4.2 Cell Culture

Human uroepithelial cell line SV‐HUC‐1 (RRID:CVCL_3798) was purchased from the American Type Culture Collection (Manassas, VA, USA) and maintained in the F‐12K medium with 10% FBS (Gibco). The BC cell line T24 (RRID: CVCL_0554), 5637 (RRID: CVCL_0126), and J82 (RRID: CVCL_0359) were acquired from the Institute of Cell Biology, Chinese Academy of Sciences (Shanghai, China). T24 and 5637 cells were cultured in RPMI 1640 supplemented with 10% FBS. J82 cells were maintained in the MEM medium with 10% FBS. All cells were grown at 37°C in a 5% CO_2_ humidified incubator. All cells underwent regular Mycoplasma screening and were verified as contamination‐free.

### 4.3 Arraystar Human 5‐methylcytosine (m^5^C) Small RNA Modification Microarray

Arraystar human m^5^C small RNA modification microarrays were purchased from Kangcheng Biotech (Shanghai, China). This procedure is similar to that described in our previous study [18]. Total RNA was extracted and then immunoprecipitated using an anti‐m^5^C antibody (Mab‐006‐500) with 1 mg Dynabeads^TM^ M‐280 anti‐mouse IgG (Invitrogen, 11201D). The modified RNAs, labeled with Cy5, were isolated from the immunoprecipitated complex using antibody‐conjugated magnetic beads as the “IP”. RNAs without m^5^C modification, labeled with Cy3, were retrieved from the supernatant and designed as “Sup”. After hybridization, scanning, and normalization, differentially expressed m^5^C‐methylated RNAs were obtained.

### 4.4 tRNA Bisulfite Sequencing

The tRNA bisulfite sequencing was conducted by CloudSeq Inc. (Shanghai, China). Total RNA was used to isolate the small RNA fraction (<200 nt) using the MirVana Isolation Kit (ThermoFisher). The enriched small RNAs underwent deaminoacylation in a solution of 0.1 m Tris–HCl (pH 9.0) and 1 mm Ethylenediaminetetraacetic Acid (EDTA) for 30 min at 37°C. Subsequently, the de‐aminoacylated RNA was subjected to bisulfite conversion and purification using an EZ RNA Methylation Kit (Zymo Research). The tRNA libraries were constructed utilizing the GenSeq Small RNA Library Prep Kit (GenSeq, Inc.), and Sanger sequencing was conducted by Ruibiotech (Guangzhou, China).

### 4.5 m^5^C Immunoprecipitation ‐3′/5′‐Adaptor Ligation Reverse Transcription Polymerase Chain Reaction (RT‐PCR)

We conducted m^5^C immunoprecipitation (IP) combined with 3′/5′‐adaptor ligation RT‐PCR to specifically detect m^5^C‐modified tsRNAs. Initial isolation of small RNAs (<200 nt) was performed with the Mirvana miRNA Isolation Kit (ThermoFisher, #AM1561). Subsequently, incubation with 5µg an m^5^C antibody(Abcam; ab10805) for 5 h at 4°C was performed. 100 µL protein A/G beads (Santa Cruz, #Sc‐2003) were then added to the mixture, followed by rotating at 4°C overnight incubation. The m^5^C modified RNAs were isolated from the beads and treated with the demethylating enzyme ALKBH3 (Solarbio, #P04974‐50µg) to remove methylation. Next, small RNAs were treated with T4 polynucleotide kinase to enable adaptor ligation to the RNA termini. Subsequently, 5′ adaptors and 3′ adaptors carrying a 5′ phosphate and a 3′ dideoxycytidine (ddC) modification were ligated to RNA ends using T4 RNA ligase1. cDNA synthesis was performed employing the RT Reagent Kit (Vazyme #R323). Finally, primers were designed target the RNA–adaptor ligation sites. We performed qPCR using Vazyme SYBR Green Master Mix (#Q711‐02). The specific sequences of the 5′‐adaptors, 3′‐adaptors, and qPCR primers are presented in Tables S1 and S2.

### 4.6 Northern Blotting and Northwestern Blotting

Small RNAs were extracted and resolved using 12% TBE‐urea PAGE for subsequent analysis, followed by transferred onto nylon membranes. Northern blotting was conducted according to previously established protocols [49]. Digoxigenin (DIG)‐labeled DNA probes were designed to hybridize with target RNAs on the membrane. After hybridization, an anti‐DIG antibody (Roche, #11093274910) was applied and allowed to bind for 2 h at 25°C. Signal detection was performed. Probe sequences are provided in Table S3.

For Northwestern blotting, membranes were blocked with 5% BSA and probed overnight at 4°C with anti‐m^5^C antibodies (Abcam; ab10805), Following washes, membranes were treated with the corresponding secondary antibody for 1 h. Protein bands were visualized using ECL Chemiluminescent Substrate reagents (Abbkine).

### 4.7 m^5^C IP Combined with Bisulfite Conversion Assay (m^5^C‐BS‐RNA)

Initially, small RNA fractions were enriched by the Mirvana Kit and then enriched with an m^5^C antibody. The samples were subsequently treated with 0.1 m Tris‐HCl (pH 9.0)/1 mM EDTA buffer, followed by bisulfite conversion and purification with the EZ RNA Methylation Kit (Zymo Research). The purified small RNAs were detected using 3′/5′‐ adaptor ligation RT‐qPCR. Finally, single‐base sequencing was used for sequence alignment. Cytosines (C) that remained unconverted to uracil (T) after bisulfite treatment were identified as m^5^C‐modified sites.

### 4.8 RNA Pull‐Down and RNA IP (RIP)

Pierce Magnetic RNA‐protein Pull‐Down Kit (Thermo Fisher Scientific, 20164) was used to conduct RNA pull‐down assays. mtRC mimics contained a site‐specific m^5^C modification at the corresponding cytosine residue, whereas unmodified tRC mimics (as a critical negative control) lacked this modification. The tRFs were synthesized and HPLC‐purified by Sangon Biotech and biotinylated using the Thermo Scientific Pierce RNA 3′ Desthiobiotinylation Kit (#20163), then captured by Streptavidin Magnetic Beads. The supernatant of the cell lysate was added to beads for pull‐down followed by mass spectrometry and WB analysis. Sequences of the mimics are listed in Table S3.

RIP assays were conducted using RIP Kit (Millipore, #17‐704) with anti‐RBM4 (Proteintech; 11614‐1‐AP) and anti‐GFP (Santa Cruz Biotechnology; sc‐9996). Finally, the extracted tRFs were analyzed by m^5^C IP ‐3′/5′‐adaptor ligation RT‐PCR.

### 4.9 Patient‐Derived Organoid Culture

BC tissues were processed, and organoid cultures were conducted following the protocol described previously [50].

### 4.10 Ubiquitination Assay

After adding MG132 (final concentration,10 µM; Sigma–Adlrich, #M8699) to cell cultures for 6 h, IP lysis buffer was used for 30 min. The lysates were immunoprecipitated overnight IP at 4°C with rotation using an anti‐RBM4 antibody (Proteintech; 11614‐1‐AP) or IgG (Proteintech; 30000‐0‐AP). To measure RBM4 ubiquitination, an antibody against ubiquitin (Cell Signaling Technology; 3936S) was used, and the immunoprecipitated proteins were quantified using Western blotting (WB).

### 4.11 Chromatin Immunoprecipitation (ChIP)

To examine the binding of H3K18la to the IL1RAP and VASH2 promoter, chromatin immunoprecipitation (ChIP) was performed using a SimpleChIP Plus Enzymatic Chromatin IP Kit (CST, 9004) following the manufacturer's protocol.

### 4.12 Extracellular Acidification Rate (ECAR) Assay

Cells (5.5 × 10^3^ /well) were placed in XFe 96‐well microplates for 24 h, followed by washing and incubation in an Agilent base medium for 1 h. ECAR was monitored using the Glycolysis Stress Test Kit and the Seahorse XFe96 Analyzer, as per the guidelines supplied by Agilent. The glycolytic activity was tested on a microplate through sequential injections of glucose (25 mm), oligomycin A (1 µm), and 2‐DG (100 mm).

### 4.13 Animal Experiments

The multi‐stage BC models induced by CdCl_2_ were established in our previous study [18].

T24 cells (2.5 × 10^6^) were subcutaneously implanted into 5‐week‐old BALB/c nude mice (Guangdong Animal Center). After 7 days, NS agomir, tRC agomir, or mtRC agomir (all synthesized by Sangon Biotech) were administered into tumors via multipoint intratumoral injections every three days, totaling five injections. Weekly tumor assessments were taken for a duration of 4 weeks. Then, tumors were removed for examination. Refer to Table S3 for the sequence listings.

All animal experiments adhered to institutional care and used guidelines, with approval from the Institutional Ethics Committee for Clinical Research and Animal Trials at the Second Affiliated Hospital (A2023‐014).

### 4.14 Human Tissue Specimens

Under the approval of the Ethics Committee of the Second Affiliated Hospital of Guangzhou Medical University (Approval No. KY‐2025‐009‐02), twelve paired BC samples and adjacent bladder tissues were collected. All patients provided written informed consent before sample acquisition. The basic demographic and clinical characteristics of bladder cancer patients and healthy individuals are shown in Tables S4 and S5, respectively.

### 4.15 Statistical Analysis

We used GraphPad Prism software (RRID:SCR_002798) for statistical analysis. Independent triplicate experiments were conducted. Values are indicated as the mean ± standard error of the mean (SEM). We used unpaired two‐tailed t‐tests or repeated‐measures ANOVA for statistical comparisons, with *P* < 0.05 considered significant. Differences among multiple groups were analyzed using one‐way ANOVA followed by Tukey's post hoc test where applicable. A linear trend analysis was performed to assess time‐dependent changes in mtRC and RBM4 expressions.

## Author Contributions

Xiaoling Ying designed and performed experiments. Yapeng Huang and Jian Huang performed animal experiments. Qinyu Cai and Danni Zhang conducted *vitro* assays. Cong Chen, Yuxi Nie, and Baotong Yang performed the RIP and ECAR assays. Chuan Li, Wenyu Hu, and Chang Xiong performed IHC assays. Chengcheng Zhang, Ding Ji, and Yaomin Liang organized the data. Weidong Ji, Wenqi Wu, and Mei Yang designed the project and provided critical feedback on the manuscript. All authors reviewed and approved the final version.

## Conflicts of Interest

The authors declare no conflicts of interest.

## Supporting information




**Supporting File**: advs74569‐sup‐0001‐SuppMat.docx.

## Data Availability

Arraystar Human m^5^C small RNA modification microarray data are available in the Gene Expression Omnibus (RRID:SCR_005012) with accession numbers GSE286269 and GSE286244. The tRNA bisulfite sequencing data can be accessed in the SRA database under the accession number PRJNA1205871. The mRNA sequencing data from this research are available in the SRA database with accession number PRJNA1196016.
