# Supplementary material for: m5C‐Modified tRF3b‐CysGCA‐23 Suppresses Bladder Cancer Malignancy by Repressing H3K18 Lactylation via Stabilizing RBM4
Source: Adv Sci (Weinh). 2026 Feb 27;13(26):e22294. doi: 10.1002/advs.202522294 (PMC13159122; doi:10.1002/advs.202522294)
Supplement: Supplementary file 1 — Supporting File: advs74569‐sup‐0001‐SuppMat.docx. [file ADVS-13-e22294-s001.docx]

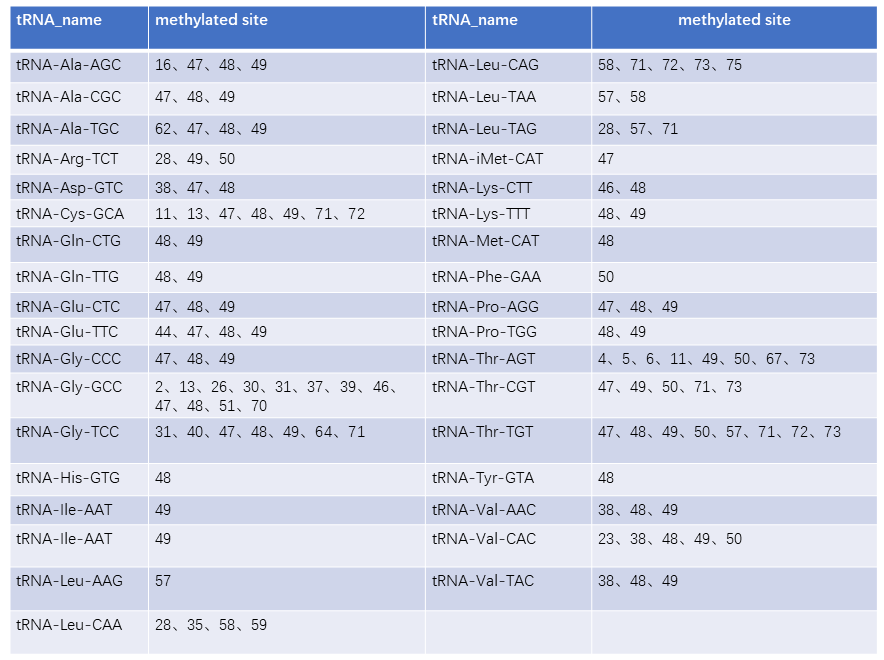


**Figure S1.** **the sites of m^5^C modification in tRNA in T24 cells using tRNA bisulfite sequencing**


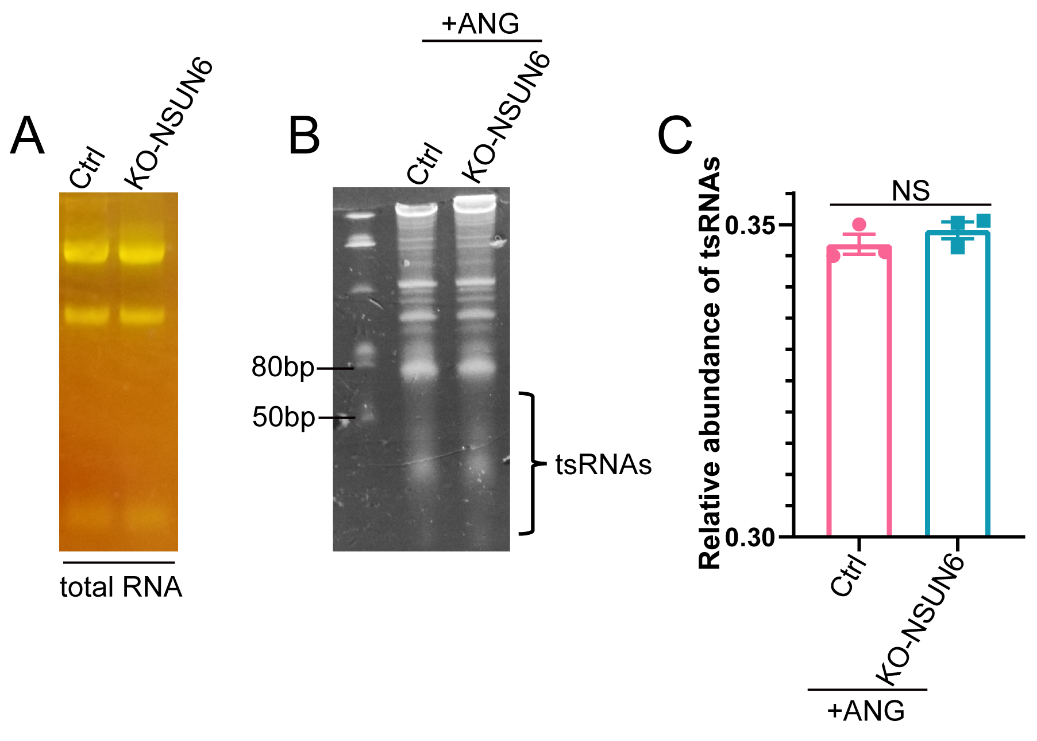


**Figure S2.** **NSUN6 does not affect angiogenin (ANG) sensitivity.** (A) Equal amounts of total RNA were isolated from NSUN6-knockdown and control cells. (B) Small RNAs were isolated from total RNAs and subjected to ANG treatment in vitro. (C) No significant differences in tRF abundance were observed between the two groups after ANG digestion.

**
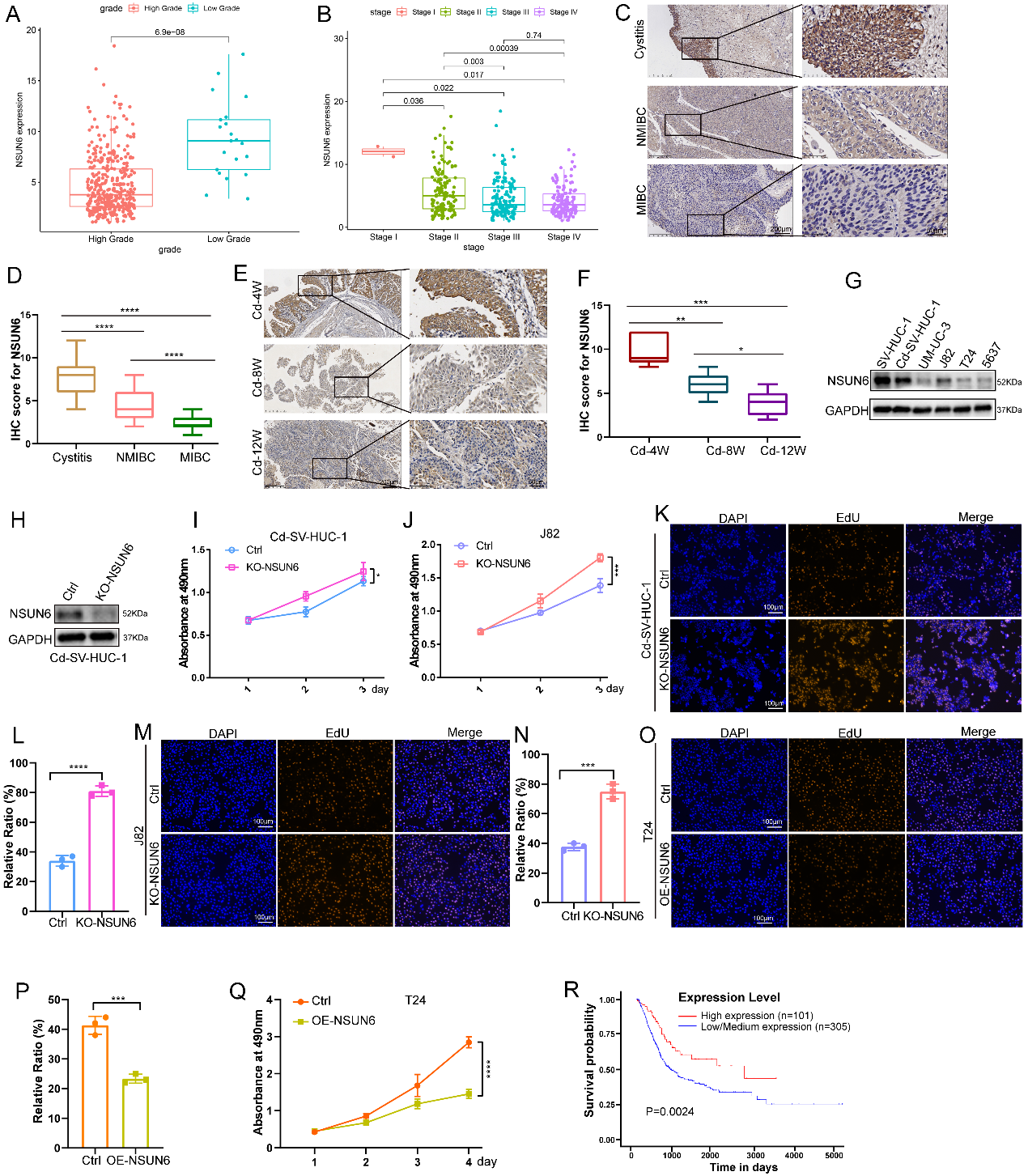
**

**Figure S3.** **NSUN6 is lowly expressed in BC and inhibits cell proliferation.** (A) Lower NSUN6 level was observed in high‐grade tumors than in low‐grade BC from the TCGA database. (B) NSUN6 expression was markedly lower in the advanced stages (stage III/IV) than that in the early stages (stage I/II). (C, D) The expression levels of NSUN6 were detected in cystitis tissues, NMIBC, and MIBC tissues exhibiting the lowest expression levels using immunohistochemistry staining. (E, F) The expression levels of NSUN6 in multi-stage bladder tissues were tested. (G) The abundance of NSUN6 was examined in SV-HUC-1, CdCl2-transformed malignant cells (Cd-SV-HUC-1) and BC cells (UM-UC-3, J82, T24, 5637) using WB. (H) The stable cells with knockout NSUN6 were constructed using the CRISPR/Cas9 gene editing technology. (I, J) CCK-8 assays demonstrated that NSUN6 depletion strongly increased Cd-SV-HUC-1 and J82 cell growth. (K-N) The results of EdU assays also revealed that NSUN6 knockdown enhances cell proliferation. (O-Q) NSUN6 overexpression suppressed the proliferation ability of T24 cells. (R) Patients with high NSUN6 revealed a dramatically longer survival time than patients with low expression. **P* < 0.05, ***P* < 0.01, ****P* < 0.001, *****P* < 0.0001.

**
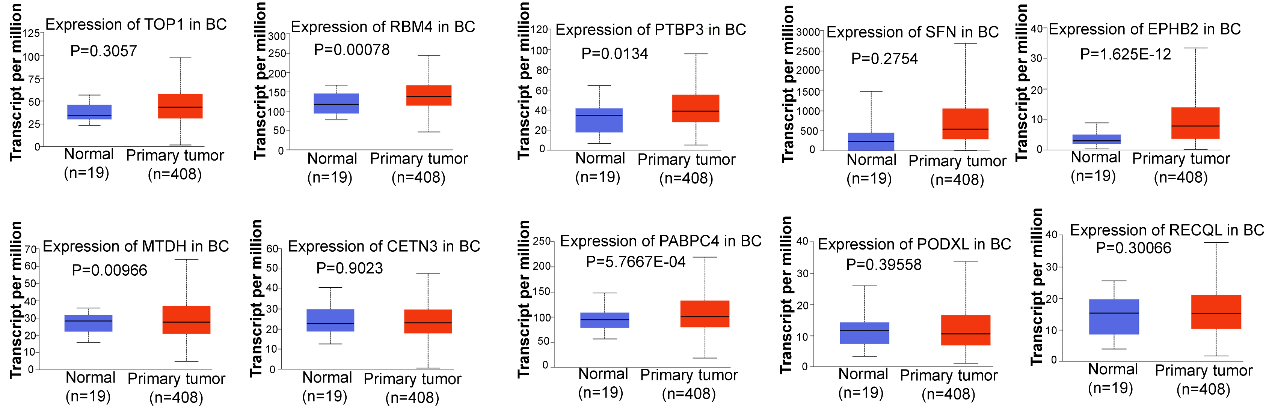
**

**Figure S4.** **The expression levels of ten genes (TOP1;** **RBM4; PTBP3; SFN；EPHB2;** **MTDH; CETN3; PABPC4; PODXL; RECQL) in TCGA.**

**
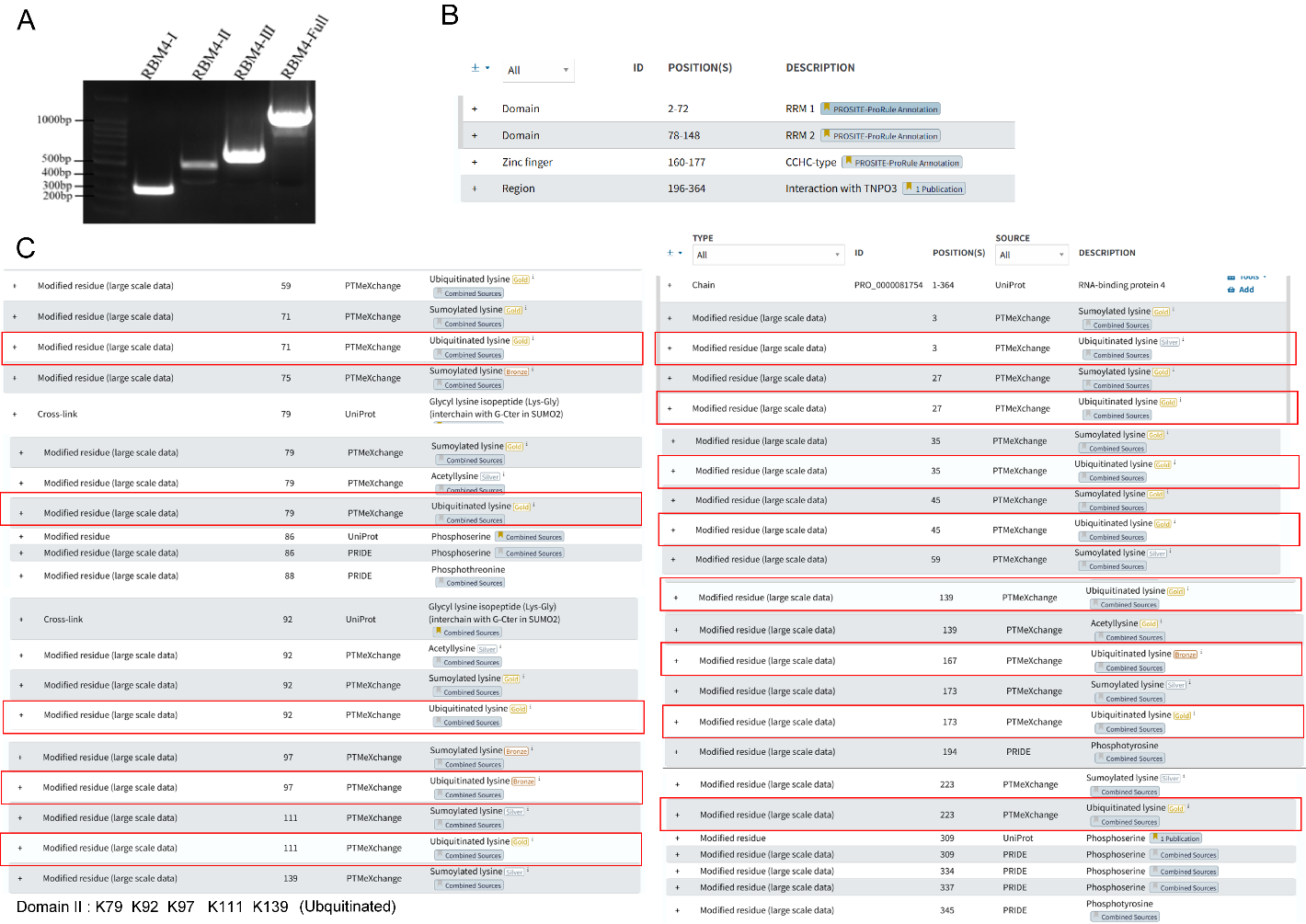
**

**Figure S5.** **Ubiquitination Site Distribution in RBM4.** (A) Construction of vectors carrying GFP-tagged full-length (domain I-IV (1-364aa)) and truncated RBM4. (B) RBM4 protein structure analysis. (C) According to UniProt annotations, ubiquitination sites of RBM4 are mainly distributed in Region I and Region II, with five ubiquitination sites located within Region II.

**
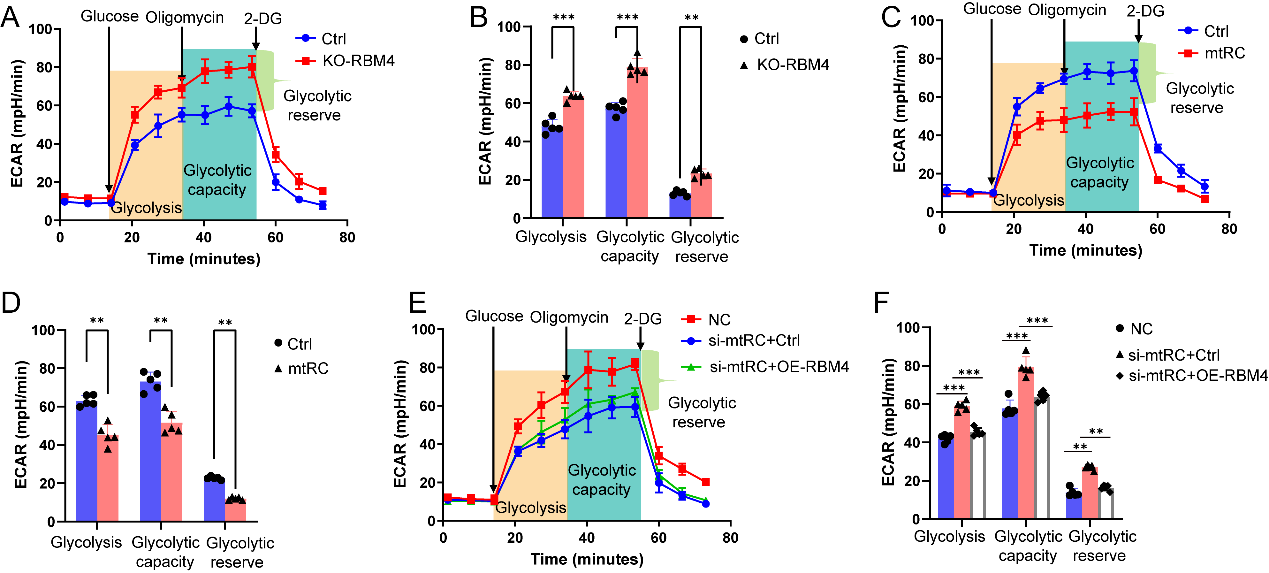
**

**Figure S6. RBM4 suppresses glycolytic capacity in BC cells.** (A, B) ECAR results revealed that RBM4 knockdown accelerated glycolysis in 5637 cells. (C, D) ECAR assays indicated that overexpression of mtRC inhibited glycolysis in 5637 cells. (E, F) ECAR experiment indicated that overexpression of RBM4 rescued glycolysis in mtRC knockdown 5637 cells. ***P* < 0.01, ****P* < 0.001.

**
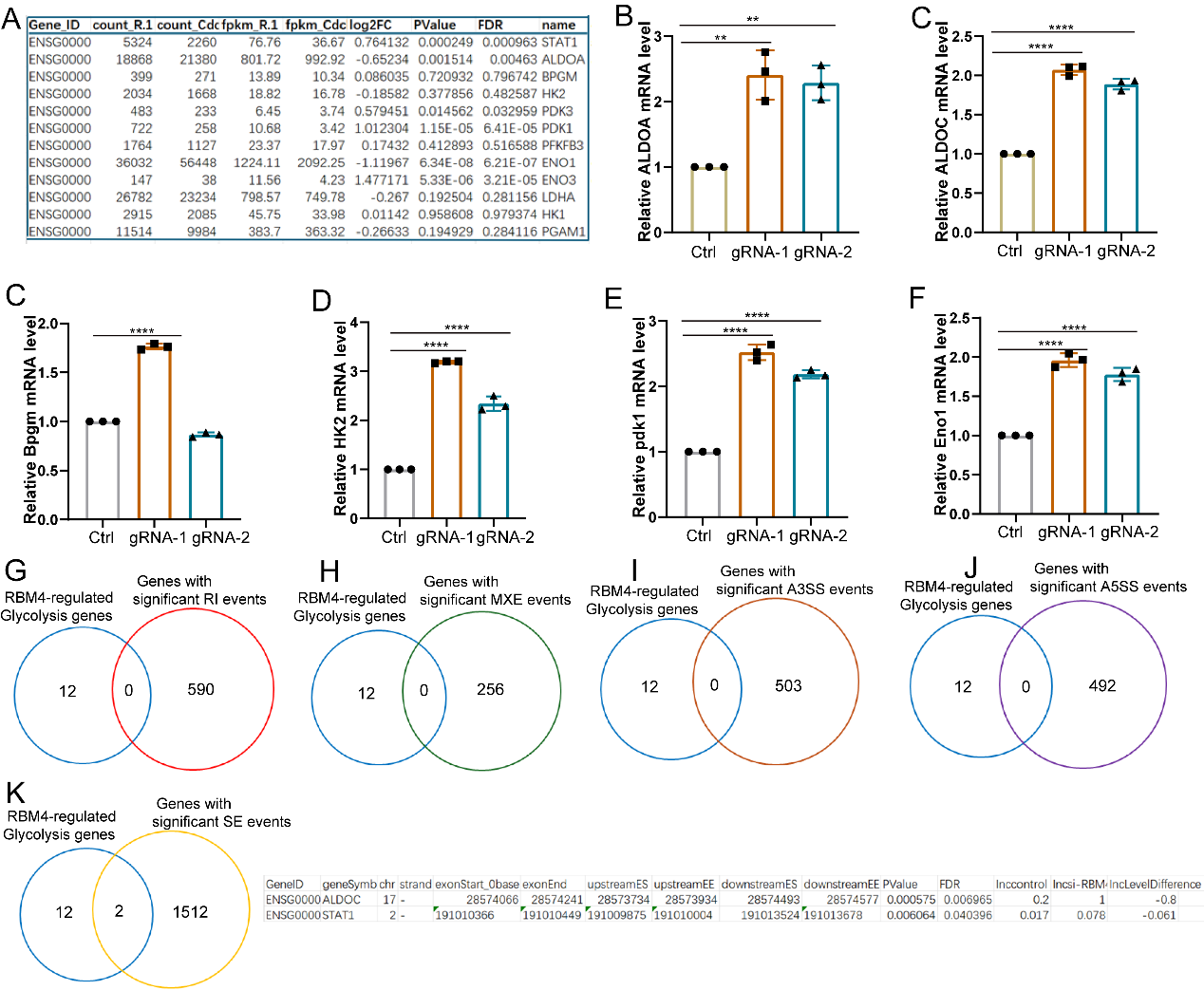
**

**Figure S7.** **RBM4 Regulation of Glycolysis-Related Gene Expression.** (A) The mRNA levels of glycolytic metabolism-related genes were detected in RBM4 knockdown cells in RNA-seq data. (B-F) The mRNA levels of glycolytic metabolism-related genes in RBM4 knockdown cells were confirmed by RT-qPCR. (G-K) Comprehensive splicing analysis of glycolysis-related genes in RBM4 knockdown and control cells.

**
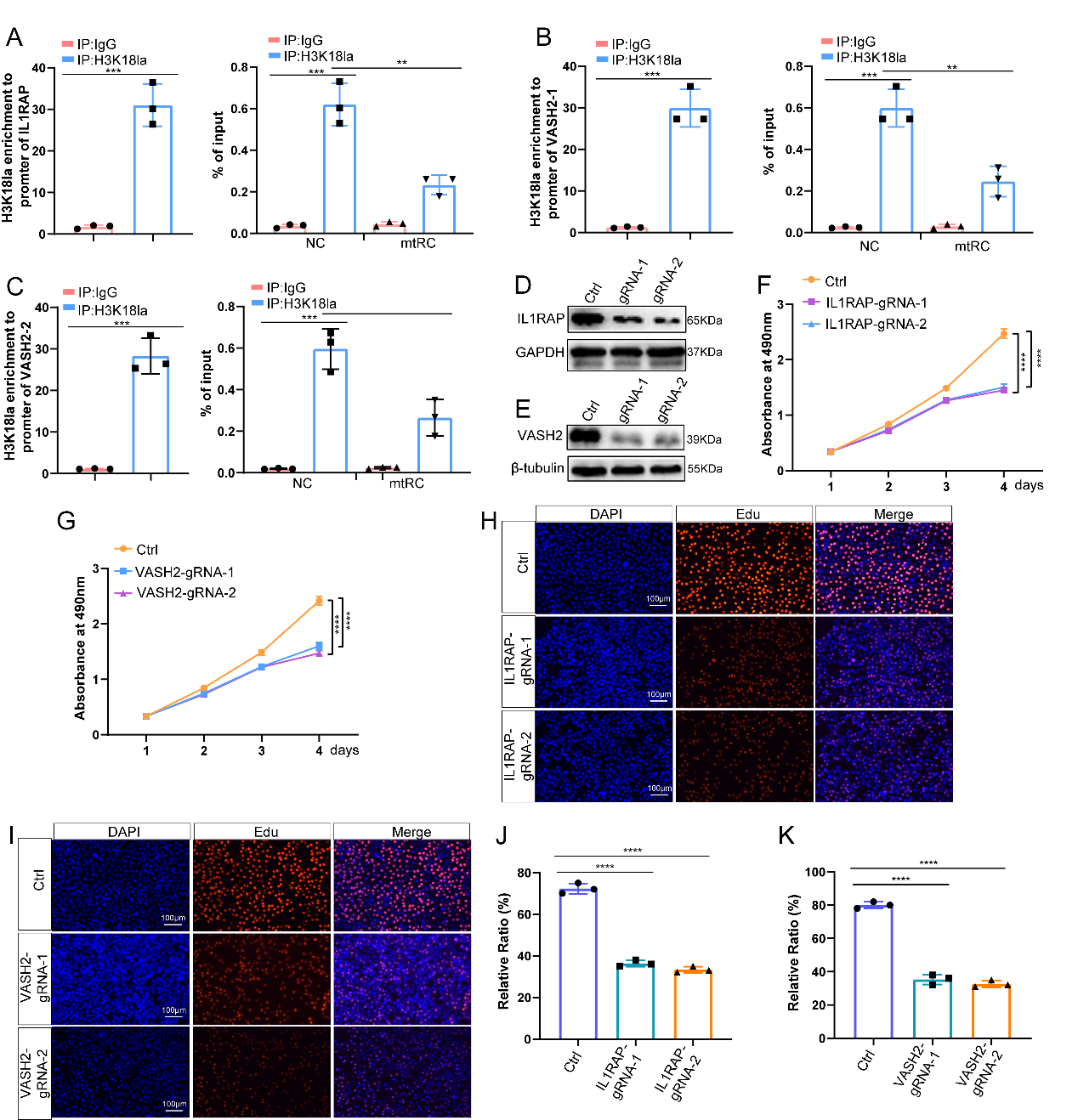
**

**Figure S8. IL1RAP and VASH2 are targets of H3K18 lactylation and function as oncogenes in BC.** (A-C) The enrichment of H3K18la at the IL1RAP and VASH2 promoter regions were assessed by ChIP-qPCR in overexpressing mtRC 5637 cells and control cells. (D, E) IL1RAP-deficient and VASH2-deficient 5637 lines were generated. (F, G) Cell viability of IL1RAP-deficient and VASH2-deficient 5637 cells were assessed using the CCK-8 assay. (H-K) Assessment of proliferation in IL1RAP-deficient and VASH2-deficient 5637 cells via EdU assay. ***P* < 0.01, ****P* < 0.001, ****P* < 0.0001.

**Supplementary Table 1.** The sequences of 5′, 3′-adaptor and qPCR primer

| **Name** | **sequence** |
| --- | --- |
| 5′- adaptor | GAUGAAAGUUCAGAGUUCUACAGUCCGACGAUC |
| 3′- adaptor | /5′Phos/ AGAUCGGAAGAGCAGUGUCGUGGUC/3′ddC/ |
| tRF3b-Cys^GCA^-23-F | TCCGACGATCTCAAATCCGG |
| tRF3b-Cys^GCA^-23-R | CTTCCGATCTTGGAGGGAGT |
| U6 Reverse | TTCACGAATTTGCGTGTCAT |
| U6 forward | CGCTTCGGCAGCACATATAC |
| RBM4 -gRNA-1 | CAAGGTGAACCACCCTCTTT |
| RBM4 -gRNA-2 | GTAATGGTGCAGGTTGCGTA |
| RBM4 -gRNA-3 | CGCTCACTCTTCGAGCAGTA |
| Plex-RBM4-F | ccgactctactagaggatccatggtgaagctgttcatcgg |
| Plex-RBM4-R | cctctagactcgagcggccgcttaaaaggctgagtaccgcg |
| NSUN6 -gRNA-1 | TAGCACGTGCACTATAACAC |
| NSUN6 -gRNA-2 | GGCGGTTAAACTTGATATGG |
| NSUN6 -gRNA-3 | CATGTTGCGTCTGGCTAATA |
| Plex-NSUN6-F | ccgactctactagaggatccatgtctattttccctaagat |
| Plex-NSUN6-R | cctctagactcgagcggccgcctatgtgcttttgcattttac |
| IL1RAP-gRNA-1 | GTGTCAAACCGACTATCACT |
| IL1RAP -gRNA-2 | GACGTACGTTTCATCTCACC |
| VASH2-gRNA-1 | AATGGCCGCTATGGCTCATT |
| VASH2 -gRNA-2 | CCGACCCAAGTGAGAAGCCG |
| ALDOC-L-F | GAGTGACCTTCCTGTCT |
| ALDOC-L-R | CTTCTCTGTAACATACTGAC |
| ALDOC-E7-F | GGCTGAGGTCTTGGCT |
| ALDOC-E7-R | CAATGCCATTCTGGGACAGCT |
| STAT1-L-F | GATGTCTCAGTGGTACGAAC |
| STAT1-L-R | ACCTTGTGCCCCAACAA |
| STAT1-E3-F | AGCAAGACTGGGATACACCA |
| STAT1-E3-R | CCGGGGAACTTTCACATATT |
| STAT1-5’UTR-F | TGCGCGCAGAAAAGTTTCATT |
| STAT1-5’UTR-R | GTGCCAAGACTGTCGAGGTT |
| STAT1-CDS-1-F | ACCTGCTCCCTCTCTGGAAT |
| STAT1-CDS-1-R | TGAATGTGATGGCCCCTTCC |
| STAT1-CDS-2-F | AAGCACCAGAGCCAATGGAA |
| STAT1-CDS-2-R | TCCTCAGGAGACATGGGGAG |
| STAT1-3’UTR-1-F | TTCCGTGGCACTGCATACAA |
| STAT1-3’UTR-1-R | ACTGAAACTTAGGTTCTCGCCA |
| STAT1-3’UTR-2-F | ACTGGCAGTTTTCCATTGGT |
| STAT1-3’UTR-2-R | TCCCCCTACCAGATCCATGA |
| STAT1-3’UTR-3-F | TGCTGTATTCTTCTTTGGTGGAGA |
| STAT1-3’UTR-3-R | ATTGTATGCAGTGCCACGGA |

**Supplementary Table 2.** The primer sequences of qPCR and siRNA

| **Name** | **sequence** |
| --- | --- |
| ALDOA-F | CATCGTGGAGCCTGAGATCC |
| ALDOA-R | CAGCACCTTCTCGGTCACAT |
| ALDOC-F | TGCCTCTAGCTGGGACTGAT |
| ALDOC-R | CACTTGGCAAAGTCAGCACC |
| Bpgm-F | GAAGCTCGGAACTGTGGGAA |
| Bpgm-R | TGGCCTAGCTCTTCCAGGAT |
| HK2-F | TCACGGAGCTCAACCATGAC |
| HK2-R | GCTCCAAGCCCTTTCTCCAT |
| PDK1-F | GCTTCATCCAGGAACATTGGC |
| PDK1-R | ATGCCGAGTCCCGCTAGA |
| Eno1-F | CCTGCCCTGGTTAGCAAGAA |
| Eno1-R | GGCGTTCGCACCAAACTTAG |
| Si LDHA | CAGGGATATTATTGACTAATAGC |
| Si LDHB | GGCAACAGTTCCAAACAATAAGA |
| Si STAT1 | CGGAACCCAGGAATCTGTCCTTCTT |
| GPR137C-F | CTCTGTGCCAGACTGTCGTC |
| GPR137C-R | GAGATATGGTGACCACCACCAA |
| IL1RAP-F | GAGCCTCATAGCTTCCAGCC |
| IL1RAP-R | TGGGTCGGAGAGTGCCTTAT |
| VASH2-F | CCCCCTGTGATCCATTCACC |
| VASH2-R | TCCACCAAACCTCATTGCGA |
| IL1RIP (chip)-F | TGGCACCGATCTCAACTGC |
| IL1RIP (chip)-R | CGGGGAAAGGAGAGGGTTAGT |
| VASH2 (chip) F-1 | GGTCACTTCTCGGGTGGTTT |
| VASH2 (chip) R-1 | AAATCCGATCGCTGTGGTGT |
| VASH2 (chip) F-2 | ACACCACAGCGATCGGATTT |
| VASH2 (chip) R-2 | CAACAAAGAGGGGCGGGTC |

**Supplementary Table 3.** The sequences of probes used in this study

| **Name** | **sequence** |
| --- | --- |
| Inhibitor-tRC/mtRC | TGGAGGGAGTAACCGGATTTGA |
| mimic-tRC | TCAAATCCGGTTACTCCCTCCA |
| mimic-mtRC | TCAAATCCGGTTACTCCC(Me)TCCA |
| Biotin-mtRC | TCAAATCCGGTTACTCCC(Me)TCCA (5'- Biotin) |
| Biotin-tRC | TCAAATCTCGCTGGGGCCTCCA (5'- Biotin) |
| Dig- tRC/mtRC | TGGAGGGAGTAACCGGATTTGA (3'- digoxigenin) |
| Dig-U6 | TGGAACGCTTCACGAATTTG (3'- digoxigenin) |
| agomir mtRC | UCAAAUCCGGUUACUCCC(Me)UCCA (2'- OMe) |
| agomir tRC | UCAAAUCCGGUUACUCCCUCCA (2'- OMe) |
| antagomir mtRC | UGGAGGGAGUAACCGGAUUUGA (2'- OMe) |
| NS agomir | CAGUACUUUUGUGUACAA (2'- OMe) |

**Supplementary Table 4. basic demographic and clinical characteristics of bladder cancer patients and healthy individuals**

| **Characteristics** | | **TNM Stage** | | | | | |
| --- | --- | --- | --- | --- | --- | --- | --- |
| Age | Gender | Tumor depth | Lymph node | Metastasis | Histological Type | Grade | Muscle Invasion Status |
| ≥60 | Male | T2 | N1 | M0 | Urothelial Tumors | High | Y |
| <60 | Male | Ta | N0 | M0 | Urothelial Tumors | Low | N |
| <60 | Male | Ta | N0 | M0 | Urothelial Tumors | Low | N |
| <60 | Male | Ta | N0 | M0 | Urothelial Tumors | Low | N |
| <60 | Female | Ta | N0 | M0 | Urothelial Tumors | Low | N |
| ≥60 | Male | T2 | N0 | M0 | Urothelial Tumors | High | Y |
| ≥60 | Male | T2 | N0 | M0 | Urothelial Tumors | High | Y |
| ≥60 | Male | T1 | N0 | M0 | Urothelial Tumors | Low | N |
| ≥60 | Male | T2 | N0 | M0 | Urothelial Tumors | High | Y |
| ≥60 | Female | T2 | N0 | M0 | Urothelial Tumors | High | Y |
| ≥60 | Female | Ta | N0 | M0 | Urothelial Tumors | Low | N |
| ≥60 | Female | T1 | N0 | M0 | Urothelial Tumors | Low | N |

**Supplementary Table 5. basic demographic of healthy individuals**

| **Characteristics** | |
| --- | --- |
| Age | Gender |
| ≥60 | Male |
| <60 | Female |
| <60 | Male |
| ≥60 | Male |
| <60 | Male |
| ≥60 | Male |
| ≥60 | Male |
| ≥60 | Male |
| ≥60 | Male |
| ≥60 | Female |
| <60 | Female |
| ≥60 | Female |

**Supplementary Materials and Methods**

**Lentiviral transduction to establish stable cell lines**

For virus transduction, 293T cells (RRID: CVCL_0063) were transfected with the appropriate lentiviral vector according to the Lipofectamine® 3000 reagent (Invitrogen) protocol. The LentiORF pLEX-MCS vector was used for overexpression, with the target plasmid and the packaging plasmids pCMV-dR8.2-dvpr (RRID: Addgene_8455) and PLP-VSVG at a 1:1:0.5 ratio. The LentiCRISPR v2 (RRID: Addgene_52961) was used for CRISPR-Cas9, with the target plasmid and the packaging plasmids PAX2 (RRID: Addgene_117398) and PLP-VSVG at a 1:1:0.5 ratio. To establish stable cell lines, target cells were transduced by using the above lentiviruses with polybrene (8 μg /ml, Sigma). After 12 h of transduction, cells were cultured further. Then the cells were selected with 1 μg /ml puromycin (Sigma) for 5 days. The sequence of gRNA and the primer are listed in Table S1.

**RNA isolation and qRT-PCR**

Total RNA was extracted using TRIzol (Invitrogen) according to the manufacturer's instructions. cDNA synthesis was performed using PrimeScript™ RT Reagent Kit with gDNA Eraser (TaKaRa). Quantitative real-time PCR (qPCR) using Fast SYBR Green PCR Master Mix (Applied Biosystems) was performed on a Step-One Fast Real-time PCR System (Applied Biosystems). All data were analysed using the 2-△△CT method.

The primer sequences are shown in Tables S1 and S2.

**Silencing and overexpression of tRFs using an inhibitor or mimic**

tsRNA mimics and inhibitors were chemically synthesized and HPLC-purified by Sangon Biotech. mtRC mimics contained a site-specific m^5^C modification at the corresponding cytosine residue, whereas unmodified tRC mimics lacked this modification. tsRNA inhibitors were synthesized with stability-enhancing backbone modifications, including phosphorothioate linkages and 2′-O-methyl modifications, and were used according to the manufacturer’s specifications. Sequences of the inhibitors and mimics are listed in Table S3. For transfection, the Lipofectamine RNA iMAX reagent was used according to the manufacturer’s instructions. RNA was collected after 48 h to detect the effects of the inhibitors or mimics.

**Western blot**

The same amount samples were run on SDS-PAGE gels and transferred onto Immobilon-P PVDF membranes (Merck Millipore). After blocking with 5% BSA in TBST for 1 h, the membranes were incubated with primary antibodies (Anti-RBM4 antibody (Proteintech, #11614-1-AP); Anti-NSUN6 antibody (Proteintech, #117240-1-AP); Anti-GFP antibody (Santa Cruz Biotechnology;,sc-9996); Anti-PTBP3 antibody (Proteintech, #14027-1-AP); Anti-EPHB2 antibody (Abcam, # ab252935); Anti-MTDH antibody (Abcam, # ab227981); Anti-PABPC4 antibody (Proteintech, #14960-1-AP); Anti-β-tubulin(Proteintech, #10094-1-AP); Anti-GAPDH antibody (Proteintech, # 10494-1-AP) ; LDHA (Proteintech # 19987-1-AP); LDHB (Proteintech, # 14824-1-AP); anti-L-Lactyl Lysine (PTM BIO, PTM-1401RM), anti-H3K18la (PTM BIO, PTM-1406RM); anti-H3K9la (PTM BIO, PTM-1419RM); anti-H4K8la (PTM BIO, PTM-1415RM); anti-H4K12la (PTM BIO, PTM-1411RM) overnight at 4 °C and then the secondary antibody for 1h. Immunoreactive bands were visualized by the Enhanced Chemiluminescence (ECL) reagents (Thermo Fisher Scientific).

**Immunofluorescence**

Cells were fixed with 4% paraformaldehyde, blocked with 1% BSA, permeabilized with 0.1% Triton X-100 for 20 min and processed for immunofluorescence. After blocking with 1% BSA in TBST for 1 h, and incubated overnight at 4 °C with anti-RBM4 antibody (Proteintech, # 60292-1-Ig) or CK5 antibody (Abcam, # ab52635), followed by incubation with Alexa Fluor™ 488 goat anti-Rabbit IgG (H+L) Cross-Adsorbed Secondary Antibody(1:1000; A-11008; Invitrogen, RRID: AB_143165), Alexa Fluor 568 donkey anti-rabbit IgG (H+L) (1:1000; A-11011; Invitrogen, RRID:AB_143157). Nuclear staining was performed using 1× 4′,6-diamidino-2-phenylindole (S2110; Solarbio, Beijing, China, RRID: AB_10638865), and images were obtained with a confocal microscope (LSM880; Carl Zeiss, Oberkochen, Germany).

**Immunohistochemistry (IHC) assay**

For IHC, human tissues were incubated at 65 °C for 30 min and then rehydrated in a gradient of ethanol. Endogenous peroxidase was blocked using 3% H_2_O_2_, followed by antigen retrieval. Thereafter, the tissues were incubated with primary antibodies (NSUN6 antibody (Proteintech, #117240-1-AP)) at 4 °C overnight after blocking with 5% BSA buffer for 30 min. A semiquantitative scoring system was used to assess IHC staining, as previously described [1].

**Nucleo-cytoplasmic separation experiment**

Nuclear and cytoplasmic fractions were separated using a nuclear and cytoplasmic extraction kit (Beyotime, P0028) according to manufacturer’s instructions. The extracted tRFs were analyzed by m^5^C immunoprecipitation (IP) -3'/5'-adaptor ligation RT-PCR.

**Cycloheximide (CHX) Chase** **Assay**

Cells were treated with 50 μM cycloheximide (CHX) and harvested at indicated time points. Protein was extracted from the cells and subjected to western blot analysis. Protein levels were measured with densitometric intensity.

**Proliferation** **and EdU** **assays**

Proliferation assays were performed as previously described [1]. Cell lines were seeded in 96-well plates and incubated for 0–5 days. Cell proliferation was tested using a CellTiter 96 AQueous One Solution Cell Proliferation Assay Kit (Promega).

BeyoClick EdU-488 Kit (Beyotime, China) and Cell Proliferation EdU Image Kit (Orange Fluorescence) (Abbkine) were used for EdU assays. Cells were cultivated in medium containing 10 μM EdU before fixing with 4% paraformaldehyde and subsequently stained with EdU reaction buffer. To visualize the DNA, the cells were stained with DAPI and observed with fluorescence microscope.

References

[1] Jin H, Ying X, Que B, Wang X, Chao Y, Zhang H, Yuan Z, Qi D, Lin S, Min W,et al. N(6)-methyladenosine modification of ITGA6 mRNA promotes the development and progression of bladder cancer. EBioMedicine. 2019; 47:195-207.
